# Emotion regulation use in daily-life and its association with success of emotion-regulation, self-efficacy, stress, and state rumination

**DOI:** 10.3389/fpsyg.2024.1400223

**Published:** 2024-10-22

**Authors:** Isabell Int-Veen, Magdalena Volz, Agnes Kroczek, Andreas J. Fallgatter, Ann-Christine Ehlis, Julian A. Rubel, David Rosenbaum

**Affiliations:** ^1^Department of Psychiatry and Psychotherapy, Tübingen Center for Mental Health (TüCMH), University Hospital of Tübingen, Tübingen, Germany; ^2^Psychotherapy Research Unit, Department of Psychology, Osnabrueck University, Osnabrueck, Germany; ^3^German Center for Mental Health (DZPG), Tübingen, Germany; ^4^LEAD Graduate School and Research Network, University of Tübingen, Tübingen, Germany

**Keywords:** ecological momentary assessment, rumination, stress, emotion regulation, daily diary

## Abstract

**Introduction:**

Investigations on emotion regulation strategies (ERS) primarily focus on the influence of instructed emotion regulation (ER) on outcomes. However, recent work has shown that selection of ERS is dependent on, e.g., situational demands and personal resources.

**Methods:**

In this current investigation, we used an online diary to investigate ERS used by free choice and their association with ER-success, stress and rumination. We identified four factors of ERS: cognitive perspective change, cognitive-behavioral problem-solving, suppression-distraction and body-social ERS. Associations of ERS with stress, state-rumination and ER-success were investigated using multilevel-mixed-models, allowing to separate within- and between-subject effects.

**Results:**

Our results show that, on a within-subject level, all adaptive ERS were positively associated with ER-success, while maladaptive ERS as well as higher stress and state rumination were negatively associated with ER-success. On the other hand, only within-subject cognitive ERS were associated with higher self-efficacy. Maladaptive ERS-use was consequently positively associated with stress and state rumination. Surprisingly, only cognitive perspective change ERS were negatively associated with state rumination. Cognitive-behavioral problem-solving was positively associated with stress and success of emotion regulation.

**Discussion:**

We interpret these results in the light of situational constraints of ERS-use and the importance of the assessment of these in future studies.

## Introduction

As soon as someone experiences emotions, one has to deal with them in one way or the other. This is also referred to as emotion regulation (ER) and has been intensely studied for the last few decades (Gross, 2014). The process model of emotion regulation (Gross, 1998, p. 275) defines ER as “[…] the processes by which individuals influence which emotions they have, when they have them, and how they experience and express these emotions. Emotion regulatory processes may be automatic or controlled, conscious or unconscious, and may have their effects at one or more points in the emotion generative process.” More specifically, there are five stages where the emotion-generative process might be altered by ER: the situation selection (e.g., avoidance), the situation modification (e.g., behavioral problem-solving), attentional deployment (e.g., distraction, rumination), cognitive change (e.g., acceptance, reappraisal) and response modulation (e.g., suppression). Initially, research was primarily focused on the use of single ER-strategies (ERS) trying to identify them and differentiate their consequences and efficacy by experimental emotion-induction (Webb et al., 2012). However, recent studies found that often multiple ERS are used concurrently or sequentially, which is also referred to as emotion-polyregulation (Aldao and Nolen-Hoeksema, 2013; Brans et al., 2013; Ford et al., 2019). Ford et al. (2019) have only recently integrated polyregulation into the process model of ER by Gross (2015).

It is important to study temporal dynamics of the involved processes in order to capture them in an ecologically valid way. In naturalistic observation studies using for instance Ecological Momentary Assessment (EMA) (Koval et al., 2020; for a comprehensive overview see Koval and Kalokerinos, 2024) and experimental studies, research has shown a crucial impact of contextual factors on ER (Kobylińska and Kusev, 2019). For example, the type and intensity of emotion (Aldao, 2013; Barrett et al., 2001; Dixon-Gordon et al., 2015), motivation (Tamir et al., 2020), timing of implementation (Diedrich et al., 2016), and perceived controllability of the situation (Troy et al., 2013) influence not only the ER-success but also ERS-choice (Koval et al., 2015; Matthews et al., 2021; Sheppes et al., 2014). Interestingly, ER-success and ERS-choice are differently influenced by psychopathology: A recent study found that strategy selection rather than the implementation of those strategies is associated with mental disorders (Houben et al., 2023).

Several strategies have been identified that are typically used in case of higher stressor intensity or more emotionally loaded situations reflected by higher negative affect (putatively maladaptive ERS, e.g., distraction, rumination) and conversely others in case of lower stressor-intensity or lower levels of negative affect (putatively adaptive ERS, e.g., acceptance, reappraisal), which is probably a consequence of decreased cognitive resources and higher cognitive load (Aldao, 2013; Barrett et al., 2001; Blanke et al., 2022; Broderick, 2005; Dixon-Gordon et al., 2015; Sheppes, 2020; Sheppes et al., 2011, 2014). Further, ERS have been found to vary in their temporal deployment, with suppression and rumination occurring more at the beginning, and reappraisal and distraction occurring more toward the end of a negative emotional episode (Kalokerinos et al., 2017).

Of particular interest for this investigation is the association of ER with rumination. Originally, rumination was seen as a cognitive vulnerability to develop depressive disorders (Nolen-Hoeksema and Morrow, 1991). It is regarded as an abstract negative thinking-style where thoughts revolve around the past while having no goal-orientation (Teismann et al., 2012). In the context of Gross’ model (2015), rumination is categorized as a form of attentional deployment where there is repetitively a passive, self-immersed focus on the emotional features and consequences of a situation. Ruminative processes and other forms of repetitive negative thought have been found not only in depression but also other psychopathologies such as anxiety, eating and substance-related disorders (Arditte et al., 2016; McLaughlin and Nolen-Hoeksema, 2011; Svaldi et al., 2012). Rumination is also observed in healthy individuals, for instance in response to stressful life events (Moberly and Watkins, 2006; Robinson and Alloy, 2003; Ruscio et al., 2015). Ruminative thinking has been found to be highly persistent by predicting future rumination, increased negative affect and depressive symptoms (Bean et al., 2020; Boemo et al., 2022; Connolly and Alloy, 2017; Kircanski et al., 2018; Moberly and Watkins, 2008; Rosenbaum et al., 2021, 2022; Rosenbaum et al., 2018a, 2018b; Ruscio et al., 2015). Interestingly, in an EMA-study by Koval et al. (2012) rumination and emotional inertia both independently predicted depression severity in healthy undergraduates as well as depressed patients.

The scope of this study was to investigate different patterns of ERS used in response to daily life stress and their association with stress-reactive rumination and ER-success and perceived self-efficacy. Ten predefined ERS were assessed in a large community sample. As the pattern of ERS use showed high interrelationships, we performed an exploratory multilevel factor analysis in order to reduce data complexity. We aimed to find patterns of which ERS are used most commonly at the same time window and how those groups were associated with ER-success (direct effect measure), self-efficacy, reduction in stress (indirect effect measure) and reduction in momentary rumination (indirect effect measure). We set up the following hypotheses: (I) We expected to find decreased ER-success in case of higher stress and higher state-rumination and higher success in case of putatively adaptive (and lower in case of maladaptive) ERS. (II) We expected to find decreased self-perceived self-efficacy in case of higher stress and higher state-rumination and higher self-efficacy in case of putatively adaptive (and lower in case of maladaptive) ERS. (III) Concerning stress, we expected to find higher stress in the evening in case of higher stress in the morning and higher rumination (Rosenbaum et al., 2022). Further, we expected to find adaptive ERS to be associated with reduced stress and rumination. (IV) Analogously, state rumination should be elevated in case of higher previous state rumination and higher stress. Likewise, we expected adaptive ERS to be negatively associated with rumination.

## Methods

### Sample

A total of 627 participants aged 18 years or older and fluent in German were recruited via flyers, emails and social media and completed the online assessment of demographic data. A total of 532 participants set up the online diary after giving written informed consent. All procedures were approved by the ethics committee at the University Hospital and University of Tübingen and in line with the Declaration of Helsinki in its latest version. After preprocessing, the final sample consisted of 144 participants with two data entries per day without day-night-shifts, ≥ 6 complete per-day data entries and no indication of careless responding (see Supplementary material S1).

### Procedure

**Figure 1:**
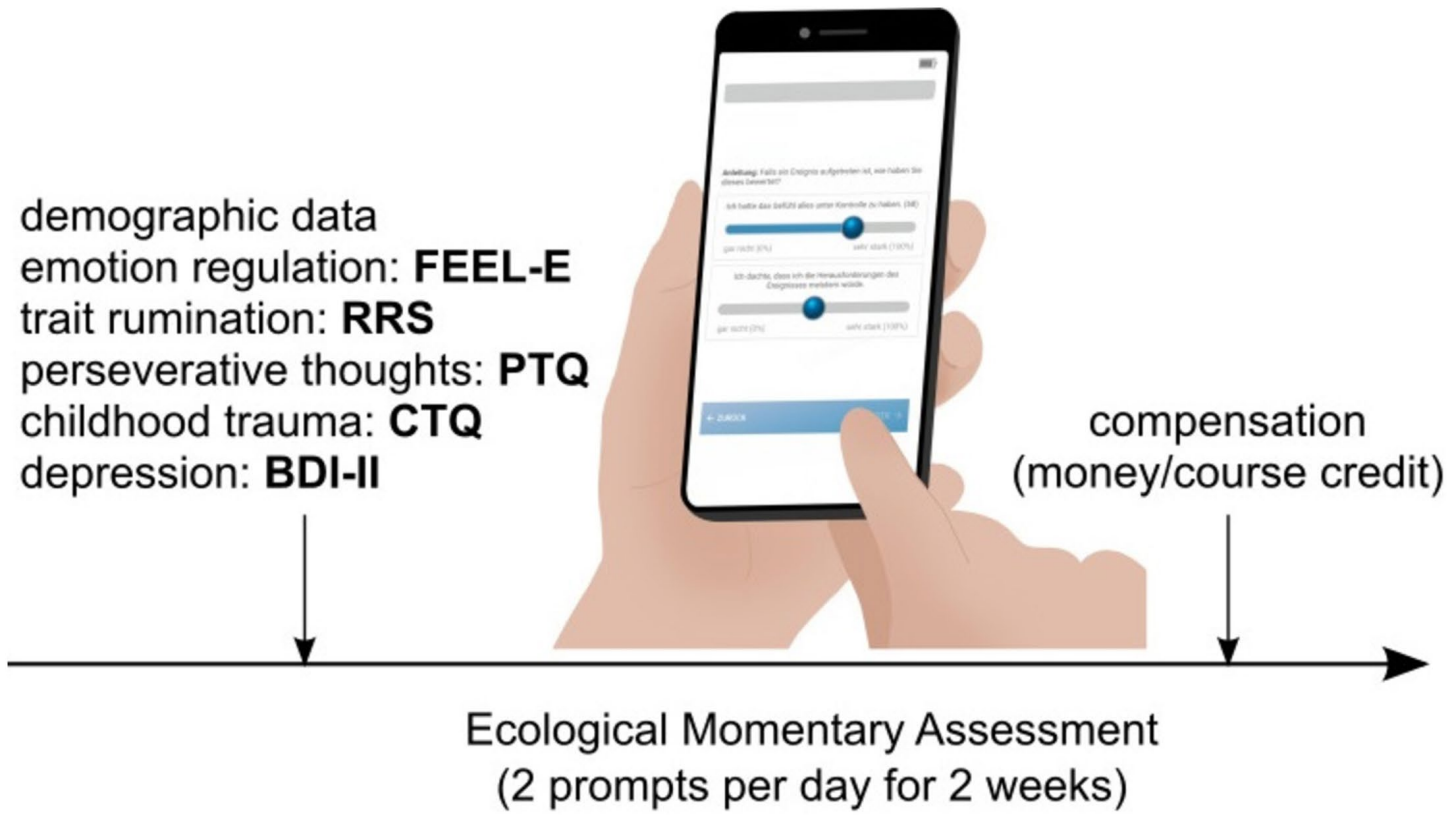
Overview over the time course of the study. FEEL-E, questionnaire assessing habitual emotion regulation; PTQ, Perseverative thinking questionnaire; RRS, Ruminative response scale; CTQ, Childhood trauma questionnaire; BDI-II, Beck depression inventory-II.

After participants received information regarding the study procedure and provided informed consent, demographic data and baseline questionnaires including a questionnaire assessing habitual ER (FEEL-E; Grob and Horowitz, 2014), as well as the Perseverative Thinking Questionnaire (PTQ; Ehring et al., 2011), Ruminative Response Scale (RRS; Nolen-Hoeksema and Morrow, 1991), Childhood Trauma Questionnaire (CTQ; Bernstein et al., 2003) and Becks Depression Inventory II (BDI-II; Hautzinger et al., 2009) were assessed. Then, participants received instructions regarding the online-diary-setup, installing it on their own smartphones using the PsyAssessor researcher-edition V2, 2019 (Machine Learning Solutions, Luxembourg). Online diary entries were assessed for 14 consecutive days where participants received emails instructing them to enter data, once at midday and once in the evening, ~5 h apart. The exact times could be freely chosen and adapted anytime. In case no data was entered within 30 min, the corresponding data point was defined as missing. At the end of the study, participants received 15€ or course credit in case they completed >50% of all data entries (see Figure 1).

### Questionnaires

#### FEEL-E

In order to assess habitual emotion regulation strategy use, participants completed the German “Fragebogen zur Erhebung der Emotionsregulation bei Erwachsenen” (FEEL-E; Grob and Horowitz, 2014). This questionnaire assesses how the corresponding person deals with different emotions (anxiety, sadness, and anger) by rating the degree to which they use the corresponding strategy in case of dealing with the specific emotion whereby two items correspond to the same strategy. Six adaptive strategies (problem-oriented, acceptance, cognitive problem solving, reevaluation, positive mood, forgetting) and six maladaptive strategies (withdrawal, self-devaluation, giving up, rumination, negative thinking, allocating blame) are rated on 5-point-Likert-Scale (1 = almost never, 5 = almost always). Like this, the FEEL-E allows to investigate emotion regulation in an emotion-specific as well as nonspecific, general way. Internal consistencies for the subscales has been proven to be high (adaptive strategies: *α* = 0.91, maladaptive strategies: *α* = 0.88). Further, test–retest-reliability after 8 months is relatively high: *r_tt_* = 0.79 for both subscales.

#### PTQ

Trait rumination was assessed using the Perseverative Thinking Questionnaire comprising 15 items (Ehring et al., 2011): “The item pool comprised three items for each of the assumed process characteristics of repetitive negative thinking: (1a) repetitive (e.g., “The same thoughts keep going through my mind again and again”), (1b) intrusive (e.g., “Thoughts come to my mind without me wanting them to”), (1c) difficult to disengage from (e.g., “I cannot stop dwelling on them”), (2) unproductive (e.g., “I keep asking myself questions without finding an answer”), (3) capturing mental capacity (e.g., “My thought prevent me from focusing on other things”).” Participants rate each item on a scale ranging from “0 = never” to “4 = almost always.” Using a non-clinical and clinical sample, the PTQ has shown to have a high internal consistency (Cronbach’s *α* > 0.94; Ehring et al., 2011) and acceptable reliability (*r_tt_* = 0.69; Ehring et al., 2011).

#### RRS

In order to assess inter-individual levels of trait rumination, the self-report Ruminative Response Scale (RRS; Nolen-Hoeksema and Morrow, 1991), a subscale of the Response Style Questionnaire (RSQ; Nolen-Hoeksema and Morrow, 1991), was used. The RRS consists of a total of 22 items which are rated on 4-point-Likert-Scales ranging from 1 = “almost never” to 4 = “almost always” and resulting in a total score ranging between 22 and 88. A high internal consistency has been observed in several studies and samples (Cronbach’s *α* > 0.88; Just and Alloy, 1997; Kasch et al., 2001; Moberly and Watkins, 2008; Nolen-Hoeksema and Morrow, 1991) including studies using the German version of the RRS (Cronbach’s *α* = 0.89–0.92; Wahl et al., 2011). Test–retest reliability, however, has been proven to fluctuate across different time spans as well as clinical and non-clinical samples: In case of non-clinical samples, test–retest reliability typically ranges between *r_tt_* = 0.80 over 6 months (Nolen-Hoeksema et al., 1994) and *r_tt_* = 0.67 over one year (Nolen-Hoeksema et al., 1999). In clinical samples, test–retest scores ranged between *r_tt_* = 0.36 over 6 months (Kasch et al., 2001), and *r_tt_* = 0.47 over one year (Just and Alloy, 1997).

#### CTQ

We used the Childhood Trauma Questionnaire (CTQ; Bernstein et al., 2003) to assess self-reported adverse childhood experiences at the age of 0–17 years. The 28-item short version of the original 70-item CTQ (Bernstein et al., 1994) comprises 25 clinical items and three validity items to screen denial (e.g., “I had the best family in the world”). The clinical items belong to five empirically derived subscales distinguishing emotional abuse, physical abuse, sexual abuse, emotional neglect and physical neglect which are summed up to the total score (range: 25–125). All statements are rated on 5-point-Likert-Scales ranging from 1 = “never true” to 5 = “very often true.” Overall, the short version of the CTQ has demonstrated satisfactorily fulfilled quality criteria. Using multiple clinical and non-clinical samples, Bernstein et al. (2003) found a consistent five-factor structure and good evidence of criterion-related validity which was evaluated by therapists’ maltreatment ratings using a structured interview, information provided by the patient as well as data of child protective investigations. Using a community sample, acceptable internal consistency of CTQ total scores (Cronbach’s *α* = 0.91) and subscale scores were observed (ranging from Cronbach’s *α* = 0.58 for physical neglect to Cronbach’s *α* = 0.94 for sexual abuse; Scher et al., 2001). Using the original version of the CTQ, high test–retest reliability values were found ranging between *r_tt_* = 0.79–0.81 (Bernstein et al., 1994; Bernstein and Fink, 1988). Similar results were replicated by various other researchers (Burns et al., 2010, 2012; Huh et al., 2017), and most importantly also using a German translation in clinical as well as non-clinical samples (Bader et al., 2009; Klinitzke et al., 2012; Wingenfeld et al., 2010).

#### BDI-II

To assess the severity of depression symptoms, we utilized the Beck Depression Inventory II, a self-report questionnaire initially developed by Beck et al. (1961) and translated into German by Hautzinger et al. (1994). Following updates in diagnostic manuals, a revised version was created by Beck et al. (1966) and translated again by Hautzinger et al. (2009). The questionnaire evaluates the presence of 21 symptoms over the past two weeks, with symptom severity quantified as a total score ranging from 0 to 63. Additionally, cut-off scores are provided to aid in interpreting these total scores. Wang and Gorenstein (2013) reported high internal consistency (Cronbach’s *α* around 0.9) and high test–retest reliability (mean interval of 2 weeks; *r_tt_* around 0.7–0.9) across various populations and languages. Notably for our study, the German version has demonstrated good ability to differentiate between depressed patients and healthy controls (Kühner et al., 2007) and is regarded as an effective screening tool for Major Depressive Disorder (Kumar et al., 2002).

### Online diary

**Figure 2:**
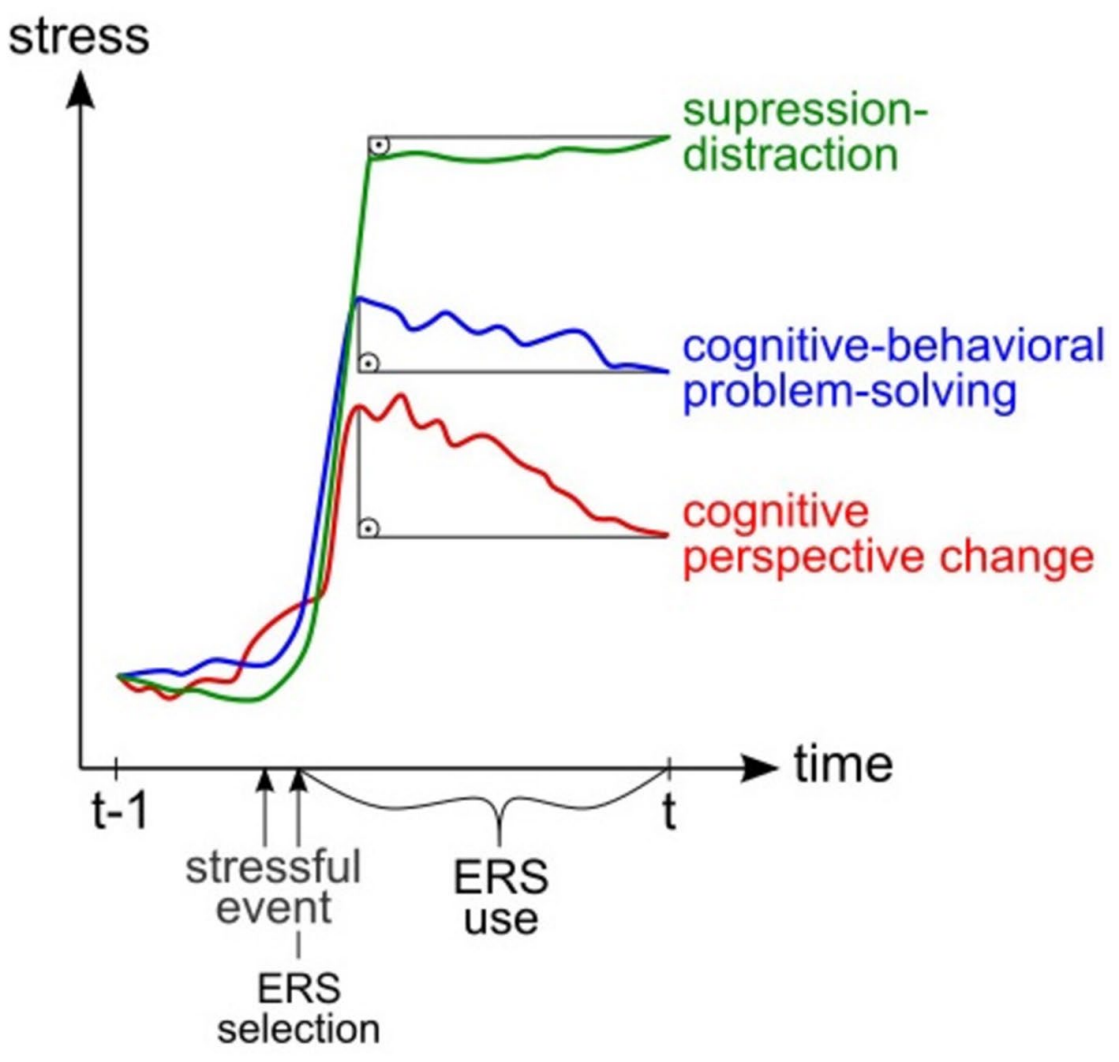
Schematic illustration of the assumed association of stress (y-axis), ER-success (displayed as rectangles) and stress-dependent ERS-use. As ERS are most probably selected in terms of situational constraints (here, different ERS are used in the case of different events), direct measures of ER-success are needed. Here, cognitive-behavioral problem-solving could be positively associated with stress in comparison to other ERS at (t) despite the undetected decline in stress after stressor-onset.

Firstly, participants stated whether something pleasant/unpleasant had happened during the past 5 h (yes/no) and could enter a free text. Using two items which were to be averaged in one factor representing self-efficacy, participants rated their agreement to the following statements on a slider (0–100%): “I felt like I was in control of everything.” and “I thought I would be able to overcome the challenges of the event.” Next, stress was assessed using a slider (0–100%) and current mood using a circumplex (arousal: aroused/relaxed, valence: positive/negative). We then assessed state rumination using three modified items of the RRS and three modified items of the Perseverative Cognitions Questionnaire (Szkodny and Newman, 2019) where participants rated their agreement using 5-point-Likert-Scales. We used this scale in another study where internal reliability was proven to be high (*α* = 0.91; Rosenbaum et al., 2022), which was replicated in the current sample (*α* = 0.89). Next, the need for ER was assessed (yes/no). Lastly participants answered whether they had applied the corresponding ERS: cognitive problem-solving, problem-oriented action/behavioral activation, reappraisal, self-compassion, acceptance, mindfulness, suppression, distraction, social support and body-based regulation. These ERS were extracted from a prior study investigating ERS-use in an open answer format qualitatively (Rosenbaum et al., 2022). To avoid ambiguities in ERS-definitions, we provided examples (see Supplementary material S2). Then, four items assessed cognitive processes and ER-success was rated (0–100%): “How successful have you been in regulating your emotions in the last 5 h?.” We chose to include this direct measure of ER-success as indirect measures such as stress and state rumination might be entangled with potential situational constraints and the regulation process itself (see Figure 2). Each diary entry took ~2 min (for all items see Supplementary material S3).

### Data preprocessing

To be able to fit cross-lagged mixed models, we excluded data entries with missing subsequent assessments and created time-lag variables for state rumination, stress and ERS-use. Participants could choose the times of data assessment freely, which is why we further removed cases where data entries were < 3 or > 18 h apart or with an alternation of day/night. We identified careless responses using the Anomaly-Case-Index-List, which reflects the unusualness of a record with respect to the group deviation it belongs to. Cases with an index >2 were removed. We further excluded participants in case there were < 6 day-complete data-entries. Lastly, in order to differentiate within- and between-person-effects of stress and state rumination, we used person-mean-centering (Enders and Tofighi, 2007; Falkenström et al., 2017; Hoffman and Stawski, 2009) while all level-2-variables were grand-mean-centered.

### Data analysis

Data analysis was done using SPSS (IBM Corp, 2019), Mplus version 8.9 (Muthén and Muthén, 2017), RStudio Version 1.4.1717 (RStudio Team, 2021) and R Version 4.0.3 (R Core Team, 2020) using the packages rmcorr (Bakdash and Marusich, 2017) for repeated-measures correlations, lme4 for mixed models (Bates et al., 2015) and lmerTest (Kuznetsova et al., 2017). As ERS-use was highly interrelated, we performed an exploratory multilevel factor analysis in Mplus in order to identify different factors. We then fitted autoregressive mixed models using maximum-likelihood-estimation in R. Please note that according to our hypotheses, we only included data entries where participants reported to have used at least one ERS. This study was not preregistered and no *a-priori* power analysis was conducted.

In order to investigate ER-success, we fitted a model with previous ER-success (t-1), current state rumination and stress as within- (WP) and between (BP)-participants factors (all variables with reference to the last 5 h). We further included the mean ERS-use of the different factors (t) (WP and BP). In a more complex model, we also added self-efficacy as WP- and BP-effects. The same models were fitted for self-efficacy instead of ER-success as dependent variable. To investigate predictors of stress at t, the basic model included previous stress (t-1) and current state rumination (t) (WP and BP) and the mean ERS-use of the different factors (t) (WP and BP). In the more complex models, we further added self-efficacy and ER-success separately as well as both at the same time. In all models, intercepts and auto-regressive effects were included as random effects allowing for heterogeneity between participants. Lastly, we fitted the analogue models for state rumination. Please note that for all dependent variables, we evaluated whether the interaction effects of stress and/or rumination explained significantly more variance compared to the most complex models without interaction effects using a Likelihood-Ratio-Test. This was never the case, which is why we report the less complex model here and the interaction-models in Supplementary material S4. In the article itself, we report the predictors of the models, as well as AIC and BIC. The correlation matrix of the predictors of the most complex model can be found in Supplementary material S5 for clarity. None of the predictors correlated above 0.49 with each other.

## Results

### Participants

On average, participants made a total of 8.51 subsequent data entries (SD = 2.25, range: 6–15; Please note that one participant entered data on 15 subsequent days) and were 24.47 years old (SD = 6.56). 90.28% of the sample were female, 9.03% male and 0.69% non-binary. 11.11% of the sample was currently/within the last 2 months in psychotherapeutic treatment (81.25% of those in treatment received (cognitive) behavioral psychotherapy, 13.50% received psychodynamic psychotherapy), while 22.92% of the total sample was diagnosed with at least one psychiatric disorder (most frequent primary diagnoses were F32 (depressive episode), *n* = 13; F50 (eating disorders), *n* = 7; F40 (phobic anxiety disorders), *n* = 6; for details see Supplementary material S6). 93.06% of the sample had never been in psychological in-patient care (0.69% once, 2.78% twice and 3.47% three or more times). The mean RRS was 31.31 (SD = 11.32), the mean BDI-II was 14.95 (SD = 10.55). Please note that this BDI-II-score is most probably biased due to the high number of females investigated (Roelofs et al., 2013).

This finding is in line with previous research investigating the BDI-II and or RRS in community samples (Economou et al., 2024; Faro and Pereira, 2020; Gomes-Oliveira et al., 2012; Roelofs et al., 2013).

### Extraction of ERS-factors

After examining the data, it became clear that most participants reported the use of several ERS at a given time point. Namely, in 18.76% of all data entries, five different ERS were used concurrently, in 18.60% three, in 17.54% four, followed by six (15.82%), two (10.03%) and seven ERS (8.97%). Only in 3.59% of all data entries only one ERS was used, followed by eight concurrently used ERS in 3.43% and nine in 3.26% of data entries. Never were all 10 ERS used at the same time point. In 54.89% of all data entries, cognitive problem-solving was used, in 42.17% acceptance, followed by behavioral problem-solving, which was used in 35.73% of all cases, self-compassion (35.32%), distraction (34.75%) and reframing (30.59%), mindfulness (27.57%), suppression (27.49%), seeking social support (24.55%) and body-based regulation, which was used in 21.78% of all cases. Therefore, we decided to reduce complexity by performing an exploratory multilevel factor analysis with Oblimin rotation in Mplus. Please note that for this analysis, only participants with WP-variation within each ERS were included (*n* = 38). According to the Scree-Plot and the Kaiser-Guttman-criterion this resulted in four factors: Factor 1 consisted of self-compassion, acceptance, reframing and mindfulness, factor 2 included cognitive and behavioral problem-solving, factor 3 included suppression and distraction and factor 4 included body-based and social regulation. We named the four factors as follows: cognitive perspective change, cognitive-behavioral problem-solving, suppression-distraction, body-social (see Supplementary material S7). The Mplus output is to be found in Supplementary material S8. We also performed a PCA using SPSS (IBM Corp, 2019, Version 28.0) where all data entries were included to identify different “clusters.” The authors are aware that PCA does not take hierarchical data into account. Nevertheless, the results replicate the findings of the Mplus analysis where only a smaller subsample could be considered. The analysis is to be found in Supplementary material S9. Note that in the free text additional ERS have been reported that are not analyzed in this work. We decided to do so as the free text format is very unspecific (e.g., with respect to adaptive/maladaptive facets of strategies like eating) and the total frequencies of those strategies were low. However, for future investigations we would like to report those free text categories that are not captured by our setup so far. The following ERS have been reported: Drinking alcohol, smoking (related, but not reported, would be consuming drugs), eating sweets, eating and vomiting, praying, sleeping/taking naps, shopping, having sex, self-harm and risk behavior, writing diaries/self-expression/art (see Supplementary material S10).

### Descriptive and correlational analysis of concurrent ERS-use

Still, ERS-factors were frequently used in combination with each other (see Supplementary materials S11, S12). Cognitive perspective change was descriptively used most frequently compared to all other factors (in a total of 1,040 data entries, see Supplementary material S11) whereas body-social ERS were used least frequently (in 819 data entries, see Supplementary material S11). While cognitive perspective change was most often used with two other factors (in 41.63% of all cases when it was used, see Supplementary material S12), namely cognitive behavioral problem-solving and suppression-distraction (see Supplementary material S10), all of the other factors were most often used with all three other factors (see Supplementary material S12). This resulted in medium positive correlations on a between-subject level among cognitive perspective change with cognitive behavioral problem-solving, *r*(142) = 0.444, *p* < 0.001, and body-social ERS, *r*(142) = 0.313, *p* < 0.001, as well as of body-social with cognitive behavioral problem-solving, *r*(142) = 0.246, *p* < 0.01 (see Supplementary material S13). On a within-person level, however, we found descriptively lower but also negative correlations (see Supplementary material S14): Cognitive perspective change was negatively associated with cognitive behavioral problem-solving, *r*(1081) = −0.069, *p* < 0.05, and with suppression-distraction, *r*(1081) = −0.098, *p* < 0.01, but positively associated with body-social ERS, *r*(1081) = 0.086, *p* < 0.01. Body-social ERS were further also negatively associated with within-person use of cognitive behavioral problem-solving, *r*(1081) = −0.128, *p* < 0.001, as well as with suppression-distraction, *r*(1081) = −0.061, *p* < 0.05 (see Supplementary material S14).

### Direct ER-success

Fitting mixed models predicting ER-success (t), higher state rumination on a WP- (t) and BP-level as well as stress (t) on a WP-level were negatively associated with ER-success. Higher state rumination and stress were associated with decreased ER-success. In case of the ERS-use factors, we observed a significant association of WP-effects for all factors: increased ER-success in case of applying cognitive perspective change, cognitive-behavioral problem-solving and body-social ERS and decreased ER-success in case of suppression-distraction. Furthermore, we observed significant BP-effects for cognitive perspective change and the use of body-social ERS indicating increased ER-success in case of individuals using cognitive perspective change and body-social ERS more often compared to others. When self-efficacy was added to the previous model, BP-stress now yielded significance and self-efficacy on a WP- and BP-level (see Table 1).

**Table 1:** Mixed models investigating predictors of ER-success_t.

	Model_1	Model_2
(Intercept)	63.32***	57.82***
	(1.23)	(2.11)
SuccessEmotionRegulation_t-1	0.04**	0.03
	(0.02)	(0.02)
WP_StateRum_t	−6.15***	−5.49***
	(0.84)	(0.84)
BP_StateRum_t	−6.05***	−5.37**
	(1.75)	(1.71)
WP_Stress_t	−0.23***	−0.23***
	(0.02)	(0.02)
BP_Stress_t	−0.11	−0.14*
	(0.07)	(0.07)
WP_PerspectiveChange_t	13.78***	12.32***
	(2.10)	(2.10)
BP_PerspectiveChange_t	9.78*	6.98
	(4.41)	(4.43)
WP_CognitiveBehavioralProblemSolving_t	8.09***	7.59***
	(1.57)	(1.55)
BP_CognitiveBehavioralProblemSolving_t	3.26	3.28
	(3.92)	(3.85)
WP_SuppressionDistraction_t	−6.71***	−6.76***
	(1.65)	(1.63)
BP_SuppressionDistraction_t	−5.67	−5.10
	(4.19)	(4.11)
WP_BodySocial_t	5.83***	5.44***
	(1.56)	(1.54)
BP_BodySocial_t	11.50**	11.07**
	(4.37)	(4.30)
WP Self-efficacy_t		0.09***
		(0.02)
BP_Self-efficacy_t		0.14***
		(0.04)
AIC	10,534.71	10,504.79
BIC	10,626.71	10,607.02
Num. obs.	1,226	1,226
Num. subjects	144	144
Var: subjects (Intercept)	65.01	69.03
Var: subjects Stress	0.00	0.00
Cov: subjects (Intercept) Stress	0.25	0.15
Var: Residual	262.87	256.40

***p < 0.001, **p < 0.01, *p < 0.05.

### Self-efficacy

We found higher previous self-efficacy (t-1) to be significantly associated with higher self-efficacy at t. Further, we observed lower previous WP-state rumination to yield a significant predictor whereas stress did not yield significance. Concerning ERS-factors we found higher WP-use of cognitive perspective change and cognitive-behavioral problem-solving to be associated with higher concurrent self-efficacy. When adding ER-success, we found WP-stress to now yield significance and the effect of WP-cognitive perspective change to diminish while ER-success was on a WP- and BP-level significantly associated with higher self-efficacy (see Table 2).

**Table 2:** Mixed models investigating predictors of self-efficacy_t.

	Model_1	Model_2
(Intercept)	34.65***	6.86
	(1.85)	(8.60)
Self-efficacy_t-1	0.25***	0.24***
	(0.03)	(0.03)
WP_StateRum_t	−6.94***	−5.54***
	(1.30)	(1.31)
BP_StateRum_t	−3.61	−0.96
	(2.89)	(2.91)
WP_Stress_t	0.03	0.08*
	(0.04)	(0.04)
BP_Stress_t	0.06	0.12
	(0.12)	(0.12)
WP_PerspectiveChange_t	16.17***	12.97***
	(3.25)	(3.27)
BP_PerspectiveChange_t	14.30	10.48
	(7.47)	(7.47)
WP_CognitiveBehavioralProblemSolving_t	5.55*	3.67
	(2.42)	(2.42)
BP_CognitiveBehavioralProblemSolving_t	4.23	1.68
	(6.59)	(6.51)
WP_SuppressionDistraction_t	0.25	1.80
	(2.55)	(2.54)
BP_SuppressionDistraction_t	−1.67	0.16
	(7.01)	(6.92)
WP_BodySocial_t	4.00	2.67
	(2.41)	(2.40)
BP_BodySocial_t	5.47	0.59
	(7.37)	(7.43)
WP_SuccessEmotionRegulation _t		0.23***
		(0.05)
BP_SuccessEmotionRegulation_t		0.43***
		(0.13)
AIC	1,1579.27	1,1554.66
BIC	1,1671.28	1,1656.89
Num. obs.	1,226	1,226
Num. subjects	144	144
Var: subjects (Intercept)	282.90	303.35
Var: subjects Stress_t-1	0.02	0.02
Cov: subjects (Intercept) Stress_t-1	−0.81	−1.20
Var: Residual	619.74	602.42

***p < 0.001, *p < 0.05.

### Stress

Fitting our models for stress, state rumination on a BP- and WP-level was positively associated with stress. On a BP-level, an increased use of cognitive perspective change was associated with lower stress while an increased use of cognitive-behavioral problem-solving was associated with significantly higher stress. On a WP-level, cognitive-behavioral problem-solving and suppression-distraction was associated with higher stress. Adding self-efficacy did not yield significance while adding ER-success was then significantly associated with higher stress on a WP- and BP-level. Further, the BP-effect of cognitive perspective change and the effect of WP-suppression-distraction no longer yielded significance. When we added ER-success and self-efficacy, WP-self-efficacy was associated with higher stress while ER-success remained negatively associated. Lastly, WP-suppression-distraction now no longer yielded significance (see Table 3).

**Table 3:** Mixed models investigating predictors of stress_t.

	Model_1	Model_2	Model_3	Model_4
(Intercept)	35.28***	35.34***	36.03***	36.09***
	(1.53)	(1.76)	(1.55)	(1.82)
Stress_t-1	0.24***	0.24***	0.22***	0.22***
	(0.03)	(0.03)	(0.03)	(0.03)
WP_StateRum_t	12.26***	12.55***	9.43***	9.89***
	(0.97)	(0.98)	(0.99)	(0.99)
BP_StateRum_t	8.19***	7.82***	6.71***	5.75**
	(1.63)	(1.70)	(1.70)	(1.75)
WP_PerspectiveChange_t	−1.43	−1.60	2.96	2.37
	(2.61)	(2.61)	(2.56)	(2.54)
BP_PerspectiveChange_t	−10.30*	−11.39*	−8.55	−9.46
	(4.56)	(4.93)	(4.65)	(4.94)
WP_CognitiveBehavioralProblemSolving_t	11.18***	11.17***	12.93***	12.80***
	(1.91)	(1.90)	(1.85)	(1.83)
BP_CognitiveBehavioralProblemSolving_t	16.63***	15.94***	17.39***	16.81***
	(3.85)	(4.08)	(3.83)	(4.03)
WP_SuppressionDistraction_t	6.04**	5.80**	3.44	3.08
	(2.03)	(2.01)	(1.98)	(1.96)
BP_SuppressionDistraction_t	7.71	8.54	7.17	7.69
	(4.27)	(4.47)	(4.26)	(4.43)
WP_BodySocial_t	−2.93	−3.06	−0.84	−1.05
	(1.93)	(1.92)	(1.88)	(1.86)
BP_BodySocial_t	−0.74	−1.12	1.54	1.82
	(4.61)	(4.89)	(4.69)	(4.95)
WP_Self-efficacy_t		0.02		0.05*
		(0.02)		(0.02)
BP_Self-efficacy_t		0.05		0.08
		(0.04)		(0.05)
WP_SuccessEmotionRegulation_t			−0.32***	−0.32***
			(0.04)	(0.03)
BP_SuccessEmotionRegulation_t			−0.18*	−0.27**
			(0.08)	(0.09)
AIC	1,0967.92	1,0985.49	1,0896.75	1,0911.38
BIC	1,1049.71	1,1077.50	1,0988.76	1,1013.61
Num. obs.	1,226	1,226	1,226	1,226
Num. subjects	144	144	144	144
Var: subjects (Intercept)	160.12	266.16	176.63	308.02
Var: subjects Stress_t-1	0.04	0.07	0.04	0.06
Cov: subjects (Intercept) Stress_t-1	−1.92	−3.51	−1.97	−3.86
Var: Residual	387.38	375.32	360.59	347.34

***p < 0.001, **p < 0.01, *p < 0.05.

### State rumination

For state rumination, current stress (WP and BP) was significantly associated with higher rumination. Furthermore, we found cognitive perspective change to be negatively associated with rumination on a WP-level. The use of suppression-distraction was positively associated with increased rumination on a WP- and BP-level. When adding self-efficacy to the model, we found a significant negative association on a WP-level while all of the other predictors remained the same. Adding ER-success also yielded significance on a WP- and BP-level. Further, we found cognitive-behavioral problem-solving to be positively associated with state rumination while WP-perspective change now no longer yielded significance. Adding self-efficacy and ER-success at the same time resulted in WP-self-efficacy, WP- and BP-ER-success yielding significance while WP-perspective change no longer yielded significance and WP-cognitive-behavioral problem-solving was now positively associated with state rumination (see Table 4).

**Table 4:** Mixed Models investigating predictors of state rumination_t.

	Model_1	Model_2	Model_3	Model_4
(Intercept)	1.29***	1.31***	1.28***	1.32***
	(0.05)	(0.05)	(0.06)	(0.05)
StateRum_t-1	0.38***	0.37***	0.37***	0.36***
	(0.03)	(0.03)	(0.03)	(0.03)
WP_Stress_t	0.01***	0.01***	0.01***	0.01***
	(0.00)	(0.00)	(0.00)	(0.00)
BP_Stress_t	0.01***	0.01***	0.01***	0.01***
	(0.00)	(0.00)	(0.00)	(0.00)
WP_PerspectiveChange_t	−0.24**	−0.18*	−0.13	−0.10
	(0.07)	(0.07)	(0.07)	(0.07)
BP_PerspectiveChange_t	−0.12	−0.09	−0.01	−0.03
	(0.16)	(0.16)	(0.17)	(0.16)
WP_CognitiveBehavioralProblemSolving_t	0.07	0.08	0.11*	0.13*
	(0.05)	(0.05)	(0.05)	(0.05)
BP_CognitiveBehavioralProblemSolving_t	−0.26	−0.26	−0.24	−0.20
	(0.14)	(0.14)	(0.15)	(0.14)
WP_SuppressionDistraction_t	0.35***	0.35***	0.29***	0.29***
	(0.06)	(0.05)	(0.05)	(0.05)
BP_SuppressionDistraction_t	0.49***	0.49***	0.38*	0.42**
	(0.14)	(0.14)	(0.15)	(0.14)
WP_BodySocial_t	−0.01	0.00	0.03	0.04
	(0.05)	(0.05)	(0.05)	(0.05)
BP_BodySocial_t	0.15	0.16	0.27	0.25
	(0.16)	(0.16)	(0.17)	(0.15)
WP_Self-efficacy_t		−0.00***		−0.00***
		(0.00)		(0.00)
BP_Self-efficacy_t		−0.00		−0.00
		(0.00)		(0.00)
WP_SuccessEmotionRegulation_t			−0.01***	−0.01***
			(0.00)	(0.00)
BP_SuccessEmotionRegulation_t			−0.01**	−0.01**
			(0.00)	(0.00)
AIC	2,332.97	2,335.14	2,304.66	2,312.19
BIC	2,414.75	2,427.15	2,396.67	2,414.42
Num. obs.	1,226	1,226	1,226	1,226
Num. subjects	144	144	144	144
Var: subjects (Intercept)	0.13	0.14	0.22	0.13
Var: subjects Stress_t-1	0.00	0.00	0.00	0.00
Cov: subjects (Intercept) Stress_t-1	−0.00	−0.00	−0.00	−0.00
Var: Residual	0.30	0.29	0.28	0.28

***p < 0.001, **p < 0.01, *p < 0.05.

## Discussion

The aim of the current online diary study was to investigate the use of emotion regulation strategies (ERS) in everyday life and their associations with perceived ER-success, self-efficacy, stress and state rumination. For this, after an assessment of baseline data, participants completed an online diary for 2 weeks. After exploration of the data and the observation of clustered use of multiple ERS at the same measurement time points, we firstly performed an exploratory multilevel factor analysis and a principal component analysis and identified four factors: cognitive perspective change, cognitive-behavioral problem-solving, suppression-distraction, body-social ERS. Although those factors already reduced data complexity, they were still rarely used alone. This underlines the importance of investigating emotion-polyregulation, which assumes that multiple ERS are implemented simultaneously/sequentially (Ford et al., 2019). Going further, this questions ERS-differentiation: our results show that ERS which were originally defined as altering different stages of the emotion-generative process (Gross, 1998), differentiated in the literature but all associated with cognitive perspective change (self-compassion, acceptance, reframing and mindfulness), loaded on one factor. Furthermore, the use of these ERS is almost always accompanied by the use of cognitive-behavioral ERS (cognitive and behavioral problem-solving). This gives rise to the idea that all of them might be facets of cognitive and behavioral ER which aims for cognitive-behavioral flexibility, for instance by cognitive perspective change. We further fitted mixed models with time-lagged variables investigating ER-success, self-efficacy, stress and state rumination dependent on ERS-factors.

Our results show that increased ER-success was significantly associated with lower stress, lower rumination, an increased use of cognitive perspective change, cognitive-behavioral problem-solving and body-social ERS. These results support our initial hypothesis (I). Interestingly, people using suppression-distraction habitually more often do not perceive their ER as less successful, however, when people use it more often than their person-mean, ER-success is perceived as less successful.

When we fitted the same models for self-efficacy as dependent variable, higher WP-rumination was associated with lower self-efficacy, which was in line with our hypothesis, whereas, contrary to our expectation, stress did not have a corresponding impact (II). We only found WP-ERS associations, namely cognitive perspective change and cognitive-behavioral problem-solving being associated with increased self-efficacy. The latter did no longer yield significance when ER-success was included. This finding suggests that the effect of ERS on self-efficacy is moderated by how successful the ER is perceived. However, the concrete causal relationship between ER-success and self-efficacy is unclear and difficult to investigate. Please note that BP-effects of ER-success and self-efficacy only correlate moderately, *r*(142) = 0.328, *p* < 0.001; however, this might also be influenced by a different item format (Likert-scale vs. slider). Disentangling these complex associations and potentially causal mechanisms will be an interesting endeavor for future research.

We further found a significant positive association of current state rumination with stress. Also, an increased WP-use of suppression-distraction was associated with higher stress. Further, individuals who use cognitive perspective change ERS habitually more often had lower stress ratings. These findings are in line with our hypotheses (III) and easily integrated into our previous findings showing an increased ER-success for cognitive perspective change as compared to the use of suppression-distraction. However, the effect of cognitive perspective change and WP-suppression-distraction no longer yielded significance when ER-success was added to the model. Higher ER-success was then associated with lower stress indicating that ER-success is more relevant than the respective strategy that was applied. However, it is important to keep in mind that there was quite some overlap between the strategies and their success. That is, cognitive perspective change was associated with more ER-success and suppression-distraction with lower success. However, in order to understand which strategies are perceived as more helpful for whom and in which situations, it still is important to get a better understanding of the effects of the different regulation strategies. When self-efficacy was added to the model, it was positively associated with stress. Please note that this seemed to be an artifact due to multicollinearity as this effect diminished when ER-success was not added to the model. In this context it is important to note that we did not randomize the order of items but it remained the same throughout the assessment. This might have an impact on how the items are answered that cannot be controlled in our analysis. Studies that randomize the order of items would not need to identify the potential effects that the order has on the data or responses to the items, but rather control for them independently.

Interestingly, despite the fact that cognitive-behavioral problem-solving was positively associated with ER-success on the WP level, we found an increased use of cognitive-behavioral problem-solving (WP and BP) to be associated with increased stress. This was originally not hypothesized but could be explained by situational demands that make the ERS-use more likely under high/low stress. In case of higher stress, cognitive-behavioral problem-solving might be used more frequently, which is why it is associated with increased stress but also increased ER-success. The stress-dependent use of ERS, namely cognitive perspective change and cognitive-behavioral problem-solving, might be explained by situational constraints such as limited cognitive resources and different needs for resources (Aldao, 2013; Barrett et al., 2001; Blanke et al., 2022; Broderick, 2005; Dixon-Gordon et al., 2015; Sheppes, 2020; Sheppes et al., 2011, 2014). As previously noted, our design does not allow us to determine whether ERS are used in a stress-dependent manner or if stress influences their use.

Please note that according to our hypotheses, we only used data entries where participants reported to have used at least one ERS when fitting our models. Additionally, we excluded participants with fewer than six consecutive data entries from the analysis to avoid distortions from less reliable subjects and to estimate parameters based on a sufficiently large data set. However, these criteria were not pre-registered. Future studies should pre-register their analyses to ensure unbiased exclusion of data points.

One question arising from these results is the definition of ER-efficacy and the appropriate measurement. This could be either done using a direct measure of ratings of ER-success or the actual difference in stress. The measure used might impact the results concerning ERS-efficiency; however, as ERS are most probably selected by situational constraints, direct measures of ER-success provide important additional information.

Investigating state rumination, we found higher stress to be associated with higher rumination on a BP- and WP-level, which supported our hypothesis (IV). Furthermore, we found a WP-increased use of cognitive perspective change to be associated with decreased state rumination. This effect, however, also diminished when ER-success was added to the model which was negatively associated with state rumination. This further resulted in WP-cognitive-behavioral problem-solving to yield a significant positive association, which also supports the idea of a substantial amount of shared variance in the model. State-of-the-art psychotherapeutic interventions already propose stressor-intensity-dependent ERS-implementation and aim to tackle ruminative processes (Goldberg et al., 2019; Hayes et al., 2007; Mennin and Fresco, 2013; Segal et al., 2008; Watkins, 2018) as they have shown to play an important role in various psychopathologies (Aldao et al., 2010). Our findings underscore the importance of rumination in everyday-life also in healthy individuals. As habitual rumination is associated with increased risk for elevated stress and negative affect, which is associated with increases in state rumination, this results in a self-sustaining loop of ruminative inertia (Bean et al., 2020; Connolly and Alloy, 2017; Kircanski et al., 2018; Moberly and Watkins, 2008; Rosenbaum et al., 2022; Ruscio et al., 2015).

The most profound limitation of the current study is the non-experimental design and correlative data which does not allow the investigation of causal mechanisms. As participants could choose ERS freely, this includes complex temporal dynamic processes of ERS selection and implementation and the reciprocal impact of situational constraints which are not controlled for in our set-up. Future studies might investigate these associations using an experimental EMA-design where participants are instructed to use ERS dependent on their current stress. Like this, it is possible to directly evaluate the situation-specificity of ER because with our data, no systematic variation of stressor-intensity has been implemented. As experimental laboratory studies with emotion-inductions often differ in terms of various crucial aspects compared to ecologically valid Ecological Momentary Assessment (EMA) data (e.g., the controllability and importance of negative events), EMA is promising for investigating these associations and it would be beneficial to combine the best of both study-designs.

A very promising and only recently developed approach is the situated assessment method (SAM^2^) (Dutriaux et al., 2023). It can be utilized to identify individual differences in habitual behavior and combined the evaluations of target behaviors and their situational influences to develop a comprehensive profile of an individual’s behavior across various situations.

Another important point to note is the very unequal sex distribution which might potentially have caused biases or make our findings less generalizable to men. First evidence regarding this comes from studies investigating coping styles and everyday stressors finding higher stress and more emotion-focused coping styles in women (Almeida and Kessler, 1998; Matud, 2004). On the other hand, however, effect sizes are low (Matud, 2004) and other studies find no gender differences (Porter et al., 2000).

Nevertheless, our results underpin the idea of situation-specific ERS-use. The present study advances the insufficiently explored interplay between emotion regulation, stress, and state rumination while emphasizing the importance of cognitive emotion regulation strategies in overcoming momentary rumination. Additionally, the findings challenge the previously distinct categorizations of emotion regulation strategies in the literature. Future research should abstain from the artificial investigation of the use of single ERS and instead focus on the use of multiple ERS in order to improve psychological theories and treatments. The categorization of cognitive ERS should be reconsidered as they might be aspects of one construct.

## Data Availability

The raw data supporting the conclusions of this article will be made available by the authors, without undue reservation.
