# Supplementary material for: Emotion regulation use in daily-life and its association with success of emotion-regulation, self-efficacy, stress, and state rumination
Source: Front Psychol. 2024 Oct 22;15:1400223. doi: 10.3389/fpsyg.2024.1400223 (PMC11534797; doi:10.3389/fpsyg.2024.1400223)
Supplement: Supplementary file 1 [file Data_Sheet_1.pdf]

Supplementary Material to:

Emotion regulation use in daily-life and its association with success of emotion-regulation, self-efficacy, stress and state rumination

Isabell Int-Veen[1*], Magdalena Volz[2], Agnes Kroczek[1], Andreas J. Fallgatter[1,3,4], Ann-Christine Ehlis[1,3,4], Julian A. Rubel[2,5], David Rosenbaum[1,5]

[1]Tübingen Center for Mental Health (TüCMH), Department of Psychiatry and Psychotherapy, University Hospital of Tuebingen, Tuebingen, Germany
[2]Psychotherapy Research Unit, Department of Psychology, Osnabrueck University, Osnabrueck, Germany
[3]German Center for Mental Health (DZPG)
[4]LEAD Graduate School & Research Network, University of Tuebingen, Tuebingen, Germany

[5]These authors contributed equally and should be regarded as joint last authors.

*Corresponding author: isabell.int-veen@med.uni-tuebingen.de

Supplementary material S1: Consort flow diagram (Moher et al., 2009).

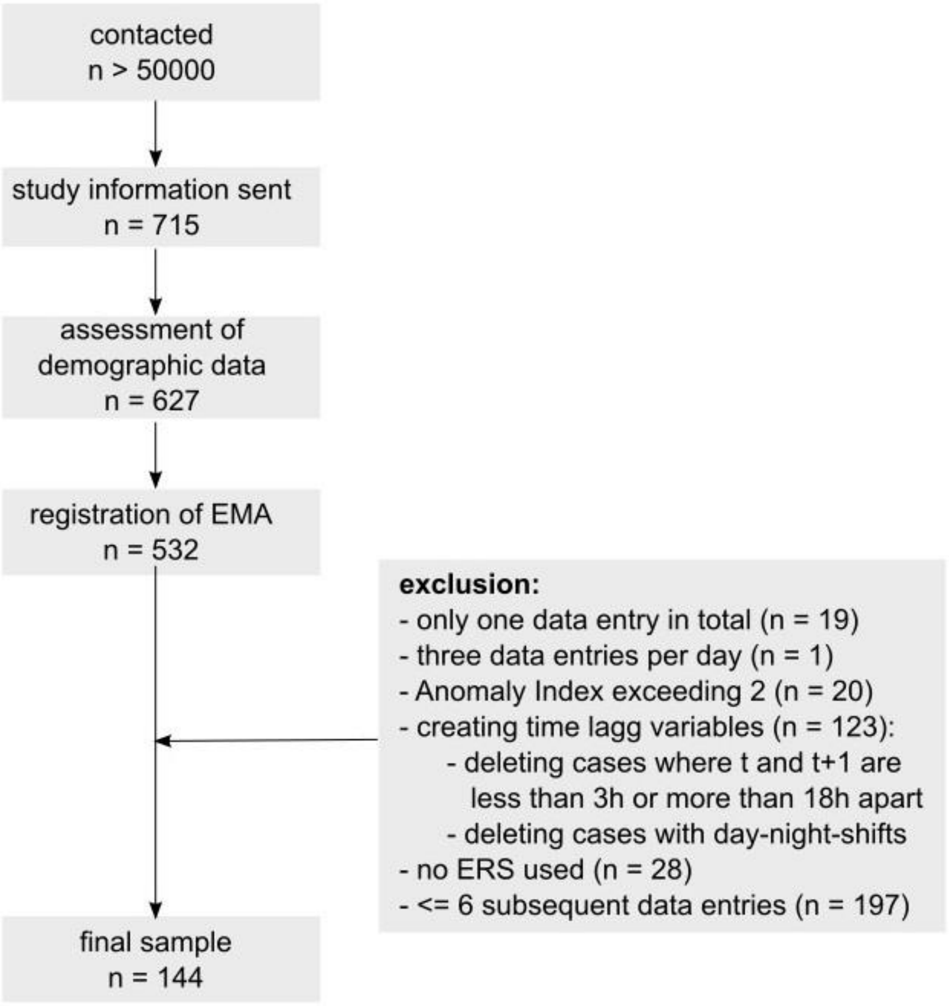

Supplementary material S2: Instructions on ERS participants received prior to data collection

English translation	German translation
After the introduction of the different answer modes, the 10 emotion regulation strategies you will be asked about in the daily surveys are explained below. In order to make the explanation as understandable as possible, the strategies are each presented using the following example.<br>*Example situation: You had a disagreement with a friend. The argument quickly picked up speed. He threw a pointed remark in your face, said he had enough for today and left the situation. You sat there alone with your feelings.*<br><br>**1. cognitive problem solving:** Here you try to find concrete solutions to acute problems by thinking. Thinking is characterized by flexible consideration of what solutions there might be and what makes sense in the long run. Different alternatives are weighed, implemented and evaluated in terms of success.<br>*Used in the above example, mental problem solving would mean briefly reflecting on the course of the dispute. What circumstances led to the situation? What goals did you have and do you have, and what goals did your friend have? How can you achieve your goals and what actions are necessary to do so? What are the obstacles and how can they be overcome?*<br><br>**2. behavioral problem-solving:** This is about the actual implementation of concrete solutions in terms of actions.<br>*Using the above example, concrete problem-oriented action could mean writing a short message to the friend and asking for a meeting to resolve the dispute.*<br><br>**3. reframing:** In re-evaluating, one tries to take different, especially more positive perspectives, for example by seeing new tasks as challenges.<br>*Using the above example, re-evaluating could mean that one does not take the friend's pointed remark personally as an act in the heat of the moment and thus feels less attacked. Or it could also mean that the argument as a whole is seen as a normal confrontation that can occur in friendships.*<br><br>**4. self-compassion:** Self-compassion is about having a positive view of oneself and valuing oneself despite a difficult situation - that is, not reacting with self-contempt or self-reproach.<br>*Applied to the above example, Self-Compassion would mean that one realizes that despite the quarrel with this friend, one is in principle a valuable and mostly lovable person - even if one certainly has one's own share in the quarrel.*	Nach der Einführung der verschiedenen Antwortmodi werden Ihnen im Folgenden die 10 Emotionsregulationsstrategien zu welchen Sie bei den täglichen Erhebungen befragt werden erklärt. Um die Erklärung möglichst verständlich zu machen, werden die Strategien jeweils an folgendem Beispiel vorgestellt.<br>*Beispielsituation: Sie hatten eine Meinungsverschiedenheit mit einem Freund. Die Auseinandersetzung nahm schnell Fahrt auf. Er warf Ihnen eine spitze Bemerkung vor dem Kopf, meinte er habe genug für heute und verließ die Situation. Sie saßen alleine mit Ihren Gefühlen da.*<br><br>**1. Gedankliches Problemlösen:** Hier versucht man durch Nachdenken konkrete Lösungen für akute Probleme zu finden. Das Denken ist gekennzeichnet durch flexibles überlegen, welche Lösungen es geben könnte und was langfristig sinnvoll ist. Verschiedene Alternativen werden abgewogen, umgesetzt und hinsichtlich des Erfolgs bewertet.<br>*Am oben genannten Beispiel eingesetzt würde gedankliches Problemlösen bedeuten, kurz den Hergang des Streits zu reflektieren. Welche Umstände haben dazu geführt, dass die Situation entstanden ist. Welche Ziele hatten und haben Sie, welche der Freund? Wie können Sie ihre Ziele erreichen und welche Handlungen sind dafür notwendig? Welche Hindernisse gibt es und wie können diese überwunden werden?*<br><br>**2. Problemorientiertes Handeln / Behaviorale Aktivierung:** Hier geht es um die tatsächliche Umsetzung von konkreten Lösungen im Sinne von Handlungen.<br>*Am obigen genannten Beispiel könnte konkretes problemorientiertes Handeln bedeuten dem Freund eine Kurznachricht zu schreiben und um ein Treffen zu bitten um den Streit zu klären.*<br><br>**3. Umbewerten:** Beim Umbewerten versucht man verschiedene, insbesondere positivere Perspektiven einzunehmen, beispielsweise indem man neue Aufgaben als Herausforderungen sieht.<br>*Am obigen Beispiel gesehen könnte eine Umbewertung bedeuten, dass man die spitze Bemerkung des Freundes als Handlung im Affekt nicht*

5. acceptance: Acceptance is about accepting the unchangeable and seeing it as part of life.
Applied to the above example, acceptance would mean that one sees the feelings that arise in the dispute in oneself - but also those that have arisen in the friend - as normal and part of life. Acceptance means accepting these feelings and continuing to search for a solution. If there is no solution, for example, if the dispute cannot be resolved, acceptance would mean accepting that as well.

6. mindfulness: Mindfulness involves trying to look at what has happened and the emotions that have been triggered in a non-judgmental way and to stay in the here and now instead of thinking about the past or worrying about the future.
Applied to the above example, a mindful attitude would mean not "getting stuck" on the argument, but focusing on the present moment. The argument has taken place. This does not need to be evaluated further in retrospect. This allows one to regain composure and possibly look for a solution later.

7. distraction: Distraction allows one to focus on other things, such as working, watching television, or engaging in some other activity.
Applied to the above example, you could conceivably listen to music to stop thinking about it for the first moment.

8. suppression: By suppressing emotions, you are willfully trying to maintain or regain control. Suppression of emotions and thoughts can be expressed in different ways, but always aims at not having to feel the emotions or think the thoughts.
Using the above example, suppression would be expressed, for example, in the fact that one tries to willfully stop thinking about the argument and to no longer feel the triggered feelings (possibly anger, rage and sadness).

9. social support: This refers to actively seeking social support. On the one hand, this can happen virtually through messenger, for example, but on the other hand through direct social contact with friends, caregivers, family or partners.
Applied to the above example, you would, for example, decide to talk to another good friend about the dispute situation, but also to ask another friend to mediate in the dispute situation.

10. body-focused regulation: Here the focus is shifted away from thoughts and emotions to the physical level. On the one hand, physical activity can be sought by doing sports or yoga, or on the other hand, explicit physical relaxation can be sought by, for example, taking a bath.
Applied to the above example, it would be conceivable that you would first go jogging to clear

persönlich nimmt und sich hierdurch weniger angegriffen fühlt. Oder auch, dass man den Streit als Ganzes als normale Auseinandersetzung sieht, die in Freundschaften vorkommen kann.

4. Self-Compassion: Bei der Self-Compassion geht es darum trotz schwieriger Situation eine positive Sichtweise auf sich selbst zu haben und sich selbst wertzuschätzen - Also nicht mit Selbstverachtung oder Selbstvorwürfen zu reagieren.
Am obigen Beispiel angewandt würde Self-Compassion bedeuten, dass man sich bewusst macht, dass man trotz des Streites mit diesem Freund prinzipiell ein wertvoller und meist liebenswürdiger Mensch ist - auch wenn man sicherlich einen eigenen Anteil an dem Streit hat.

5. Akzeptanz: Bei der Akzeptanz geht es darum, Unveränderbares annehmend hinzunehmen und als Teil des Lebens zu sehen.
Am obigen Beispiel angewandt würde Akzeptanz bedeuten, dass man die Gefühle, welche im Streit bei einem selbst auftauchen - aber auch jene die beim Freund aufgetaucht sind - als normal und Bestandteil des Lebens sieht. Akzeptanz bedeutet diese Gefühle anzunehmen und weiter nach einer Lösung zu suchen. Gibt es keine Lösung, beispielsweise wenn der Streit nicht geklärt werden kann, würde Akzeptanz bedeuten, auch dies anzunehmen.

6. Achtsamkeit: Bei der Achtsamkeit wird versucht Geschehenes und ausgelöste Emotionen wertungsfrei zu betrachten und im Hier und Jetzt zu bleiben, anstatt über Vergangenes nachzudenken oder sich um Zukünftiges zu sorgen.
Am obigen Beispiel angewandt würde eine achtsame Haltung bedeuten, nicht an dem Streit "hängen zu bleiben", sondern sich auf den jetzigen Moment zu konzentrieren. Der Streit hat stattgefunden. Dies muss im Nachhinein nicht weiter bewertet werden. So kann man wieder zur Ruhe kommen und später womöglich nach einer Lösung suchen.

7. Ablenken: Durch Ablenkung konzentriert man sich auf andere Dinge, beispielsweise indem man arbeitet, fernsieht oder einer anderen Beschäftigung nachgeht.
Am obigen Beispiel angewandt wäre es denkbar, dass Sie Musik hören, um für den ersten Moment nicht weiter darüber nachdenken zu müssen.

your head and to let off steam.

8. Unterdrücken: Indem man Emotionen unterdrückt, versucht man willentlich die Kontrolle zu behalten bzw. wiederzuerlangen. Die Unterdrückung von Gefühlen und Gedanken kann sich auf verschiedene Weise äußern, zielt jedoch immer darauf ab die Emotionen nicht spüren zu müssen bzw. die Gedanken nicht denken zu müssen.
Am obigen Beispiel angewandt würde sich Unterdrücken beispielsweise darin äußern, dass man versucht willentlich nicht mehr an den Streit zu denken und die ausgelösten Gefühle (möglicherweise Ärger, Wut und Traurigkeit) nicht mehr zu fühlen.

9. Soziale Unterstützung: Hierbei ist gemeint, dass aktiv nach sozialer Unterstützung gesucht wird. Dies kann einerseits beispielsweise virtuell geschehen durch Messenger, andererseits aber durch direkten sozialen Kontakt mit Freunden, Bezugspersonen, Familie oder Partnern.
Am obigen Beispiel angewandt würden Sie sich beispielsweise dazu entscheiden mit einer anderen guten Freundin über die Streitsituation zu sprechen, aber auch einen anderen Freund um eine Vermittlung in der Streitsituation zu bitten.

10. Körperbetonte Regulation: Hier wird der Fokus weg von Gedanken und Emotionen auf die körperliche Ebene verlagert. Einerseits kann die körperliche Aktivität gesucht werden, indem Sport getrieben wird, Yoga gemacht wird, oder andererseits die explizite körperliche Entspannung gesucht wird, indem beispielsweise ein Bad genommen wird.
Am obigen Beispiel angewandt wäre es denkbar, Sie würden erstmal joggen gehen, um den Kopf frei zu bekommen und sich auszupowern.

S3: Items of the Momentary Assessment

Items assessing previous events

English translation	German translation
Has something pleasant happened to you in the past 5 hours?	Ist Ihnen in den vergangenen 5 Stunden etwas Angenehmes im Alltag widerfahren?
Has something unpleasant happened to you in the past 5 hours?	Ist Ihnen in den vergangenen 5 Stunden etwas Unangenehmes im Alltag widerfahren?
In case yes: Which events have occurred?	Falls ja: Welche Ereignisse sind aufgetreten?

Items assessing the cognitive appraisal of these events

English translation	German translation
I felt like I was in control of everything.	Ich hatte das Gefühl alles unter Kontrolle zu haben.
I thought I would be able to overcome the challenges of the event.	Ich dachte, dass ich die Herausforderungen des Ereignisses meistern würde.

Item assessing current stress

English translation	German translation
How stressed have you been in the past 5 hours?	Wie sehr waren Sie in den letzten 5 Stunden gestresst?

Item assessing current affect

English translation	German translation
Please indicate how you have felt during the last 5 hours. Please include the strength and duration of the emotion: e.g .: Have you felt moderately sad throughout = a little unhappy, have you felt moderately sad for an hour = slightly unhappy	Bitte geben Sie an, wie Sie sich in den letzten 5 Stunden gefühlt haben. Beziehen Sie die Stärke und Dauer der Emotion mit ein: z.B.: Haben Sie sich durchgehend moderat traurig gefühlt = etwas unglücklich, haben Sie sich eine Stunde moderat traurig gefühlt = leicht unglücklich

Items assessing ruminative processes

English translation	German translation
I repeatedly thought about things that are long gone*	Ich dachte wiederholt über Dinge nach, die längst vorbei waren.
It is hard for me to let go of a thought once it has entered my head*	Ich fand es schwierig einen Gedanken loszulassen, wenn er einmal in meinem Kopf war.
I repeatedly play back past events in my mind*	Ich spielte wiederholt vergangene Geschehnisse in meinem Kopf durch.
I thought about all my shortcomings, failings and mistakes°	Ich dachte an all meine Misserfolge, Defizite und Macken.

I asked myself why I have problems that others do not have°	Ich fragte mich, warum ich Probleme habe, die andere nicht haben.
I thought about why I can´t handle things better°	Ich dachte darüber nach, warum ich die Dinge nicht besser in den Griff bekomme.

Items assessing the use of emotion regulation strategies

English translation	German translation
Have you regulated your emotions in the past 5 hours?	Haben Sie in den vergangenen 5 Stunden Ihre Emotionen reguliert?
Problem solving (e.g. searching for concrete solutions, thinking in small steps)	Gedankliches Problemlösen (z.B. konkret, lösungsorientiert in kleinen Schritten nach Lösungen suchen)
Problem-oriented action / behavioral activation (e.g. solving the problem through concrete actions; e.g. learning in case you are afraid of an exam or having a clarifying conversation in case of a dispute)	Problemorientiertes Handeln / Behaviorale Aktivierung (z.B. das Problem durch konkrete Handlungen lösen; z.B. bei Angst vor einer Prüfung lernen oder bei Streit ein klärendes Gespräch führen)
Reappraisal (take another, more positive perspective, e.g. seeing a problem as a challenge with the opportunity to grow)	Umbewerten (verschiedene positivere Perspektiven einnehmen, z.B. ein Problem als Herausforderung sehen an der man wachsen kann)
Self-compassion (e.g. having a positive view of yourself despite difficult situations, appreciating yourself)	Self-Compassion (z.B. trotz schwieriger Situationen eine positive Sichtweise auf sich selbst haben, sich selbst wertschätzen)
Acceptance (e.g. accepting the unchangeable and seeing it as part of life)	Akzeptanz (z.B. Unveränderbares annehmend hinnehmen und es als Teil des Lebens sehen)
Mindfulness (e.g. trying to be non-judgemental about what has happened and to stay in the here and now)	Achtsamkeit (z.B. versuchen Geschehenes wertungsfrei zu betrachten und im Hier und Jetzt sein)
Distraction (focus on something else, e.g. watch TV)	Ablenken (sich auf etwas anderes konzentrieren z.B. fernsehen)
Suppression (willingly suppressing emotional reactions, e.g. pull yourself together and not showing how you are doing)	Unterdrücken – Willentliche Kontrolle (emotionale Reaktionen willentlich unterdrücken, z.B. die “Zähne zusammenbeißen” und nicht zeigen wie es einem geht)
Social support (e.g. talking to a friend or parents about it or getting help from acquaintances)	Soziale Unterstützung (z.B. mit einem Freund oder den Eltern darüber sprechen oder sich von Bekannten helfen lassen)
Body-based regulation (e.g. relaxation, doing sports, taking a bath)	Körperbetonte Regulation (z.B. Entspannung, Sport machen, ein Bad nehmen)
Did you use any other strategy not listed above? If yes, which:	Haben Sie eine andere Strategie angewandt, die oben nicht aufgeführt wurde? Falls ja, welche:

Items assessing cognitive processes

English translation	German translation
My thoughts were solution-oriented.	Ich dachte lösungsorientiert.
I thought about possible solutions in small steps.	Ich dachte in kleinen Schritten über Lösungswege nach.
I thought flexibly about different aspects of the problem.	Ich dachte flexibel über verschiedene Aspekte des Problems nach.
I thought about what to do next.	Ich dachte daran, was ich konkret als Nächstes tun kann.

Item assessing success of emotion regulation

English translation	German translation
How successful have you been in regulating your emotions in the last 5 hours?	Wie erfolgreich ist Ihnen die Emotionsregulation in den letzten 5 Stunden insgesamt gelungen?

Note.* = modified items of the Perseverative Cognitions Questionnaire (Szkodny and Newman, 2019), ° = modified items of the Ruminative Response Scale (Nolen-Hoeksema, 1991).

Supplementary material S4. Models including interaction effects with ERS scales

Models investigating Success of Emotion Regulation

	Model 1	Model 2	Model 3	Model 4
(Intercept)	57.82 ***<br>(2.11)	57.54 ***<br>(2.21)	57.68 ***<br>(2.06)	57.82 ***<br>(2.11)
SuccessEmotionRegulation_t-1	0.03<br>(0.02)	0.03<br>(0.02)	0.03 *<br>(0.02)	0.03<br>(0.02)
WP_Self-efficacy t	0.09 ***<br>(0.02)	0.09 ***<br>(0.02)	0.09 ***<br>(0.02)	0.09 ***<br>(0.02)
BP_Self-efficacy t	0.14 ***<br>(0.04)	0.14 ***<br>(0.04)	0.14 ***<br>(0.04)	0.14 ***<br>(0.04)
WP_StateRum_t	-5.49 ***<br>(0.84)	-5.15 ***<br>(0.84)	-5.35 ***<br>(0.84)	-5.49 ***<br>(0.84)
BP_StateRum_t	-5.37 **<br>(1.71)	-5.67 **<br>(1.92)	-5.22 **<br>(1.62)	-5.37 **<br>(1.71)
WP_Stress_t	-0.23 ***<br>(0.02)	-0.23 ***<br>(0.02)	-0.23 ***<br>(0.02)	-0.23 ***<br>(0.02)
BP_Stress_t	-0.14 *<br>(0.07)	-0.14 *<br>(0.07)	-0.15 *<br>(0.07)	-0.14 *<br>(0.07)
WP_PerspectiveChange_t	12.32 ***<br>(2.10)	12.30 ***<br>(2.08)	12.27 ***<br>(2.11)	12.32 ***<br>(2.10)
BP_PerspectiveChange_t	6.98<br>(4.43)	6.55<br>(4.64)	6.16<br>(4.27)	6.98<br>(4.43)
WP_CognitiveBehavioralProblemSolving_t	7.59 ***<br>(1.55)	7.44 ***<br>(1.54)	7.72 ***<br>(1.56)	7.59 ***<br>(1.55)
BP_CognitiveBehavioralProblemSolving_t	3.28<br>(3.85)	2.94<br>(4.02)	4.27<br>(3.79)	3.28<br>(3.85)
WP_SuppressionDistraction_t	-6.76 ***<br>(1.63)	-6.67 ***<br>(1.62)	-6.86 ***<br>(1.64)	-6.76 ***<br>(1.63)
BP_SuppressionDistraction_t	-5.10<br>(4.11)	-4.41<br>(4.32)	-5.28<br>(3.90)	-5.10<br>(4.11)
WP_BodySocial_t	5.44 ***<br>(1.54)	5.49 ***<br>(1.53)	5.62 ***<br>(1.55)	5.44 ***<br>(1.54)
BP_BodySocial_t	11.07 **<br>(4.30)	11.78 **<br>(4.46)	12.08 **<br>(4.22)	11.07 **<br>(4.30)
WP_StateRum_t:WP_PerspectiveChange_t		9.26 **<br>(3.51)		
BP_StateRum_t:BP_PerspectiveChange_t		-3.19<br>(7.92)		
WP_StateRum_t:WP_CognitiveBehavioralProblemSolving_t		-3.69<br>(2.58)		
BP_StateRum_t:BP_CognitiveBehavioralProblemSolving_t		-4.25<br>(6.59)		
WP_StateRum_t:WP_SuppressionDistraction_t		0.22<br>(2.74)		
BP_StateRum_t:BP_SuppressionDistraction_t		1.94<br>(6.26)		
WP_StateRum_t:WP_BodySocial_t		4.27<br>(2.73)		
BP_StateRum_t:BP_BodySocial_t		8.89<br>(7.85)		
WP_Stress_t:WP_PerspectiveChange_t			0.32 **<br>(0.10)	
BP_Stress_t:BP_PerspectiveChange_t			-0.22<br>(0.25)	
WP_Stress_t:WP_CognitiveBehavioralProblemSolving_t			-0.01<br>(0.08)	
BP_Stress_t:BP_CognitiveBehavioralProblemSolving_t			-0.19<br>(0.22)	
WP_Stress_t:WP_SuppressionDistraction_t			0.05	

			(0.08)	
BP_Stress_t:BP_SuppressionDistraction_t			0.14	
			(0.26)	
WP_Stress_t:WP_BodySocial_t			-0.08	
			(0.07)	
BP_Stress_t:BP_BodySocial_t			0.10	
			(0.25)	
AIC	10504.79	10505.43	10510.07	10504.79
BIC	10607.02	10648.55	10653.19	10607.02
Log Likelihood	-5232.39	-5224.71	-5227.03	-5232.39
Num. obs.	1226	1226	1226	1226
Num. subjects	144	144	144	144
Var: Subjects (Intercept)	69.03	76.75	49.65	69.03
Var: Subjects Stress_t-1	0.00	0.00	0.00	0.00
Cov: Subjects (Intercept) Stress_t-1	0.15	0.11	0.23	0.15
Var: Residual	256.40	251.73	257.67	256.40

Models investigating Self-Efficacy

	Model 1	Model 2	Model 3	Model 4
(Intercept)	6.86<br>(8.60)	5.56<br>(8.88)	4.56<br>(8.62)	4.48<br>(8.73)
SelfEfficacy_t-1	0.24 ***<br>(0.03)	0.23 ***<br>(0.03)	0.24 ***<br>(0.03)	0.24 ***<br>(0.03)
WP_ SuccessEmotionRegulation_t	0.23 ***<br>(0.05)	0.24 ***<br>(0.05)	0.23 ***<br>(0.05)	0.24 ***<br>(0.05)
BP_ SuccessEmotionRegulation_t	0.43 ***<br>(0.13)	0.46 ***<br>(0.13)	0.47 ***<br>(0.13)	0.47 ***<br>(0.13)
WP_StateRum_t	-5.54 ***<br>(1.31)	-5.53 ***<br>(1.33)	-5.53 ***<br>(1.32)	-5.60 ***<br>(1.33)
BP_StateRum_t	-0.96<br>(2.91)	-0.49<br>(3.33)	-0.81<br>(2.88)	-0.42<br>(3.29)
WP_Stress_t	0.08 *<br>(0.04)	0.09 *<br>(0.04)	0.08 *<br>(0.04)	0.09 *<br>(0.04)
BP_Stress_t	0.12<br>(0.12)	0.11<br>(0.12)	0.16<br>(0.12)	0.13<br>(0.12)
WP_PerspectiveChange_t	12.97 ***<br>(3.27)	13.10 ***<br>(3.26)	12.93 ***<br>(3.28)	13.06 ***<br>(3.28)
BP_PerspectiveChange_t	10.48<br>(7.47)	11.93<br>(7.78)	10.21<br>(7.50)	11.15<br>(7.68)
WP_CognitiveBehavioralProblemSolving_t	3.67<br>(2.42)	3.43<br>(2.41)	3.55<br>(2.43)	3.49<br>(2.43)
BP_CognitiveBehavioralProblemSolving_t	1.68<br>(6.51)	0.60<br>(6.78)	-2.04<br>(6.67)	-0.93<br>(6.91)
WP_SuppressionDistraction_t	1.80<br>(2.54)	1.51<br>(2.53)	2.17<br>(2.55)	1.86<br>(2.56)
BP_SuppressionDistraction_t	0.16<br>(6.92)	1.44<br>(7.29)	-0.38<br>(6.93)	1.07<br>(7.30)
WP_BodySocial_t	2.67<br>(2.40)	2.27<br>(2.39)	2.37<br>(2.41)	1.99<br>(2.40)
BP_BodySocial_t	0.59<br>(7.43)	-0.77<br>(7.74)	-0.03<br>(7.63)	-0.77<br>(7.86)
WP_StateRum_t:WP_PerspectiveChange_t		-9.76<br>(5.49)		-11.11<br>(6.01)
BP_StateRum_t:BP_PerspectiveChange_t		22.41<br>(12.97)		25.81<br>(15.02)
WP_StateRum_t:WP_CognitiveBehavioralProblemSolving_t		-5.96<br>(4.05)		-4.85<br>(4.40)
BP_StateRum_t:BP_CognitiveBehavioralProblemSolving_t		-0.16<br>(10.99)		-2.03<br>(11.32)
WP_StateRum_t:WP_SuppressionDistraction_t		-8.75 *<br>(4.27)		-7.94<br>(4.75)
BP_StateRum_t:BP_SuppressionDistraction_t		-0.58<br>(10.44)		2.51<br>(11.03)
WP_StateRum_t:WP_BodySocial_t		-3.67<br>(4.26)		-7.09<br>(4.66)
BP_StateRum_t:BP_BodySocial_t		-1.07<br>(13.09)		-8.59<br>(14.33)
WP_Stress_t:WP_PerspectiveChange_t			-0.07<br>(0.16)	0.07<br>(0.17)
BP_Stress_t:BP_PerspectiveChange_t			0.13<br>(0.45)	-0.35<br>(0.54)
WP_Stress_t:WP_CognitiveBehavioralProblemSolving_t			-0.13<br>(0.13)	-0.08<br>(0.14)
BP_Stress_t:BP_CognitiveBehavioralProblemSolving_t			0.31<br>(0.38)	0.30<br>(0.41)
WP_Stress_t:WP_SuppressionDistraction_t			-0.16<br>(0.12)	-0.06<br>(0.13)
BP_Stress_t:BP_SuppressionDistraction_t			-0.40<br>(0.46)	-0.33<br>(0.51)
WP_Stress_t:WP_BodySocial_t			0.13<br>(0.11)	0.22<br>(0.13)
BP_Stress_t:BP_BodySocial_t			0.42<br>(0.45)	0.57<br>(0.51)
AIC	11554.66	11512.71	11572.40	11529.05

BIC	11656.89	11655.83	11715.52	11713.07
Log Likelihood	-5757.33	-5728.35	-5758.20	-5728.53
Num. obs.	1226	1226	1226	1226
Num. subjects	144	144	144	144
Var: Subjects (Intercept)	303.35	347.40	289.45	295.99
Var: Subjects Stress_t-1	0.02	0.02	0.01	0.01
Cov: Subjects (Intercept) Stress_t-1	-1.20	-1.53	-0.98	-0.98
Var: Residual	602.42	597.25	608.00	605.77

Models investigating Stress

	Model 1	Model 2
(Intercept)	36.09 ***	36.49 ***
	(1.82)	(1.60)
Stress_t-1	0.22 ***	0.22 ***
	(0.03)	(0.03)
WP_StateRum_t	9.89 ***	9.59 ***
	(0.99)	(1.01)
BP_StateRum_t	5.75 **	7.52 ***
	(1.75)	(1.91)
WP_SelfEfficacy_t	0.05 *	0.05 *
	(0.02)	(0.02)
BP_SelfEfficacy_t	0.08	0.08
	(0.05)	(0.04)
WP_ SuccessEmotionRegulation_t	-0.32 ***	-0.33 ***
	(0.03)	(0.04)
BP_ SuccessEmotionRegulation_t	-0.27 **	-0.22 **
	(0.09)	(0.08)
WP_PerspectiveChange_t	2.37	2.48
	(2.54)	(2.57)
BP_PerspectiveChange_t	-9.46	-9.88 *
	(4.94)	(4.74)
WP_CognitiveBehavioralProblemSolving_t	12.80 ***	12.84 ***
	(1.83)	(1.85)
BP_CognitiveBehavioralProblemSolving_t	16.81 ***	16.52 ***
	(4.03)	(3.88)
WP_SuppressionDistraction_t	3.08	3.59
	(1.96)	(1.98)
BP_SuppressionDistraction_t	7.69	7.54
	(4.43)	(4.36)
WP_BodySocial_t	-1.05	-0.88
	(1.86)	(1.87)
BP_BodySocial_t	1.82	1.45
	(4.95)	(4.73)
WP_StateRum_t:WP_PerspectiveChange_t		3.56
		(4.27)
BP_StateRum_t:BP_PerspectiveChange_t		8.07
		(7.86)
WP_StateRum_t:WP_CognitiveBehavioralProblemSolving_t		-0.26
		(3.11)
BP_StateRum_t:BP_CognitiveBehavioralProblemSolving_t		-8.63
		(6.61)
WP_StateRum_t:WP_SuppressionDistraction_t		2.19
		(3.32)
BP_StateRum_t:BP_SuppressionDistraction_t		-7.21
		(6.29)
WP_StateRum_t:WP_BodySocial_t		9.37 **
		(3.31)
BP_StateRum_t:BP_BodySocial_t		-2.38
		(7.93)
AIC	10911.38	10865.80
BIC	11013.61	11008.92
Log Likelihood	-5435.69	-5404.90
Num. obs.	1226	1226
Num. subjects	144	144
Var: Subjects (Intercept)	308.02	172.94
Var: Subjects Stress_t-1	0.06	0.03
Cov: Subjects (Intercept) Stress_t-1	-3.86	-1.84
Var: Residual	347.34	359.56

Models investigating state rumination

	Model 1	Model 2
(Intercept)	1.32 ***	1.30 ***
	(0.05)	(0.06)
StateRum_t-1	0.36 ***	0.36 ***
	(0.03)	(0.03)
WP_Stress_t	0.01 ***	0.01 ***
	(0.00)	(0.00)
BP_Stress_t	0.01 ***	0.01 ***
	(0.00)	(0.00)
WP_SelfEfficacy_t	-0.00 ***	-0.00 ***
	(0.00)	(0.00)
BP_SelfEfficacy_t	-0.00	-0.00
	(0.00)	(0.00)
WP_ SuccessEmotionRegulation_t	-0.01 ***	-0.01 ***
	(0.00)	(0.00)
BP_ SuccessEmotionRegulation_t	-0.01 **	-0.01 **
	(0.00)	(0.00)
WP_PerspectiveChange_t	-0.10	-0.10
	(0.07)	(0.07)
BP_PerspectiveChange_t	-0.03	-0.01
	(0.16)	(0.18)
WP_CognitiveBehavioralProblemSolving_t	0.13 *	0.12 *
	(0.05)	(0.05)
BP_CognitiveBehavioralProblemSolving_t	-0.20	-0.23
	(0.14)	(0.15)
WP_SuppressionDistraction_t	0.29 ***	0.29 ***
	(0.05)	(0.05)
BP_SuppressionDistraction_t	0.42 **	0.38 *
	(0.14)	(0.16)
WP_BodySocial_t	0.04	0.04
	(0.05)	(0.05)
BP_BodySocial_t	0.25	0.27
	(0.15)	(0.18)
WP_Stress_t:WP_PerspectiveChange_t		-0.00
		(0.00)
BP_Stress_t:BP_PerspectiveChange_t		-0.00
		(0.01)
WP_Stress_t:WP_CognitiveBehavioralProblemSolv-ing_t		0.00
		(0.00)
BP_Stress_t:BP_CognitiveBehavioralProblemSolv-ing_t		-0.00
		(0.01)
WP_Stress_t:WP_SuppressionDistraction_t		0.00
		(0.00)
BP_Stress_t:BP_SuppressionDistraction_t		0.00
		(0.01)
WP_Stress_t:WP_BodySocial_t		0.00
		(0.00)
BP_Stress_t:BP_BodySocial_t		0.01
		(0.01)
AIC	2312.19	2393.15
BIC	2414.42	2536.27
Log Likelihood	-1136.09	-1168.57
Num. obs.	1226	1226
Num. subjects	144	144
Var: Subjects (Intercept)	0.13	0.20
Var: Subjects Stress_t-1	0.00	0.00
Cov: Subjects (Intercept) Stress_t-1	-0.00	-0.00
Var: Residual	0.28	0.27

Supplementary material S5: Correlation matrix of predictors

Correlation Matrix of Predictors Model 2: ER-success_t

Predictor	SuccessEmotionRegulation_t-1	WP_StateRum_t	BP_StateRum_t	WP_Stress_t	BP_Stress_t	WP_PerspectiveChange_t	BP_PerspectiveChange_t	WP_CognBehProblemSolving_t	BP_CognBehProblemSolving_t	WP_SuppressionDistraction_t	BP_SuppressionDistraction_t	WP_BodySocial_t	BP_BodySocial_t	WP Self-efficacy_t	BP Self-efficacy_t
SuccessEmotionRegulation_t-1	1.00	-0.05	-0.02	-0.07	0.02	0.02	0.23	-0.05	0.17	0.04	-0.02	-0.01	0.16	0.09	0.21
WP_StateRum_t	-0.05	1.00	0.00	0.41	0.00	-0.14	0.00	0.07	0.00	0.27	0.00	-0.04	0.00	-0.19	0.00
BP_StateRum_t	-0.02	0.00	1.00	0.00	0.49	0.00	-0.09	0.00	-0.03	0.00	0.39	0.00	0.12	0.00	-0.06
WP_Stress_t	-0.07	0.41	0.00	1.00	0.00	-0.10	0.00	0.17	0.00	0.17	0.00	-0.06	0.00	-0.06	0.00
BP_Stress_t	0.02	0.00	0.49	0.00	1.00	0.00	-0.04	0.00	0.28	0.00	0.31	0.00	0.12	0.00	0.07
WP_PerspectiveChange_t	0.02	-0.14	0.00	-0.10	0.00	1.00	0.00	-0.07	0.00	-0.10	0.00	0.09	0.00	0.17	0.00
BP_PerspectiveChange_t	0.23	0.00	-0.09	0.00	-0.04	0.00	1.00	0.00	0.47	0.00	0.05	0.00	0.35	0.00	0.26
WP_CognBehProblemSolving_t	-0.05	0.07	0.00	0.17	0.00	-0.07	0.00	1.00	0.00	-0.13	0.00	0.05	0.00	0.05	0.00
BP_CognBehProblemSolving_t	0.17	0.00	-0.03	0.00	0.28	0.00	0.47	0.00	1.00	0.00	-0.01	0.00	0.27	0.00	0.19
WP_SuppressionDistraction_t	0.04	0.27	0.00	0.17	0.00	-0.10	0.00	-0.13	0.00	1.00	0.00	-0.06	0.00	-0.06	0.00

BP_Suppression-Distraction_t	-0.02	0.00	0.39	0.00	0.31	0.00	0.05	0.00	-0.01	0.00	1.00	0.00	0.18	0.00	0.01
WP_BodySocial_t	-0.01	-0.04	0.00	-0.06	0.00	0.09	0.00	0.05	0.00	-0.06	0.00	1.00	0.00	0.07	0.00
BP_BodySocial_t	0.16	0.00	0.12	0.00	0.12	0.00	0.35	0.00	0.27	0.00	0.18	0.00	1.00	0.00	0.14
WP Self-efficacy_t	0.09	-0.19	0.00	-0.06	0.00	0.17	0.00	0.05	0.00	-0.06	0.00	0.07	0.00	1.00	0.00
BP Self-efficacy_t	0.21	0.00	-0.06	0.00	0.07	0.00	0.26	0.00	0.19	0.00	0.01	0.00	0.14	0.00	1.00

Correlation Matrix of Predictors Model 2: self-efficacy_t

Predictor	Self-efficacy_t-1	WP_StateRum_t	BP_StateRum_t	WP_Stress_t	BP_Stress_t	WP_PerspectiveChange_t	BP_PerspectiveChange_t	WP_CognBehProblemSolving_t	BP_CognBehProblemSolving_t	WP_SuppressionDistraction_t	BP_SuppressionDistraction_t	WP_BodySocial_t	BP_BodySocial_t	WP_SuccessEmotionRegulation _t	BP_SuccessEmotionRegulation _t
(Intercept)															
Self-efficacy_t-1	1.00	-0.02	0.05	-0.02	0.14	-0.01	0.18	-0.01	0.10	0.03	0.02	0.01	0.08	0.01	0.14
WP_StateRum_t	-0.02	1.00	0.00	0.41	0.00	-0.14	0.00	0.07	0.00	0.27	0.00	-0.04	0.00	-0.38	0.00
BP_StateRum_t	0.05	0.00	1.00	0.00	0.49	0.00	-0.09	0.00	-0.03	0.00	0.39	0.00	0.12	0.00	-0.37
WP_Stress_t	-0.02	0.41	0.00	1.00	0.00	-0.10	0.00	0.17	0.00	0.17	0.00	-0.06	0.00	-0.39	0.00
BP_Stress_t	0.14	0.00	0.49	0.00	1.00	0.00	-0.04	0.00	0.28	0.00	0.31	0.00	0.12	0.00	-0.27
WP_PerspectiveChange_t	-0.01	-0.14	0.00	-0.10	0.00	1.00	0.00	-0.07	0.00	-0.10	0.00	0.09	0.00	0.24	0.00
BP_PerspectiveChange_t	0.18	0.00	-0.09	0.00	-0.04	0.00	1.00	0.00	0.47	0.00	0.05	0.00	0.35	0.00	0.33
WP_CognBehProblemSolving_t	-0.01	0.07	0.00	0.17	0.00	-0.07	0.00	1.00	0.00	-0.13	0.00	0.05	0.00	0.08	0.00
BP_CognBehProblemSolving_t	0.10	0.00	-0.03	0.00	0.28	0.00	0.47	0.00	1.00	0.00	-0.01	0.00	0.27	0.00	0.19

Predictor	Self-efficacy_t-1	WP_StateRum_t	BP_StateRum_t	WP_Stress_t	BP_Stress_t	WP_PerspectiveChange_t	BP_PerspectiveChange_t	WP_CognBehProblemSolving_t	BP_CognBehProblemSolving_t	WP_SuppressionDistraction_t	BP_SuppressionDistraction_t	WP_BodySocial_t	BP_BodySocial_t	WP_SuccessEmotionRegulation _t	BP_SuccessEmotionRegulation _t
WP_SuppressionDistraction_t	0.03	0.27	0.00	0.17	0.00	-0.10	0.00	-0.13	0.00	1.00	0.00	-0.06	0.00	-0.25	0.00
BP_SuppressionDistraction_t	0.02	0.00	0.39	0.00	0.31	0.00	0.05	0.00	-0.01	0.00	1.00	0.00	0.18	0.00	-0.19
WP_BodySocial_t	0.01	-0.04	0.00	-0.06	0.00	0.09	0.00	0.05	0.00	-0.06	0.00	1.00	0.00	0.15	0.00
BP_BodySocial_t	0.08	0.00	0.12	0.00	0.12	0.00	0.35	0.00	0.27	0.00	0.18	0.00	1.00	0.00	0.22
WP_SuccessEmotionRegulation _t	0.01	-0.38	0.00	-0.39	0.00	0.24	0.00	0.08	0.00	-0.25	0.00	0.15	0.00	1.00	0.00
BP_SuccessEmotionRegulation _t	0.14	0.00	-0.37	0.00	-0.27	0.00	0.33	0.00	0.19	0.00	-0.19	0.00	0.22	0.00	1.00

Correlation Matrix of Predictors Model 4: stress_t

Predictor	Stress_t-1	WP_StateRum_t	BP_StateRum_t	WP_Per-spec-tiveChange_t	BP_Per-spec-tiveChange_t	WP_Cogn-BehProb-lemSol-ving_t	BP_Cogn-BehProb-lemSol-ving_t	WP_Supp-ression-Distrac-tion_t	BP_Supp-ression-Distrac-tion_t	WP_Bo-dySocial_t	BP_Bo-dySocial_t	WP Self-efficacy_t	BP Self-efficacy_t	WP_Suc-cessEmoti-onRegula-tion _t	BP_Suc-cessEmoti-onRegula-tion _t
Stress_t-1	1.00	0.09	0.24	-0.03	0.04	0.00	0.15	0.01	0.20	-0.04	0.10	-0.04	0.00	-0.09	-0.09
WP_StateRum_t	0.09	1.00	0.00	-0.14	0.00	0.07	0.00	0.27	0.00	-0.04	0.00	-0.19	0.00	-0.38	0.00
BP_StateRum_t	0.24	0.00	1.00	0.00	-0.09	0.00	-0.03	0.00	0.39	0.00	0.12	0.00	-0.06	0.00	-0.37
WP_Per-spec-tiveChange_t	-0.03	-0.14	0.00	1.00	0.00	-0.07	0.00	-0.10	0.00	0.09	0.00	0.17	0.00	0.24	0.00
BP_Per-spec-tiveChange_t	0.04	0.00	-0.09	0.00	1.00	0.00	0.47	0.00	0.05	0.00	0.35	0.00	0.26	0.00	0.33
WP_Cogn-BehProb-lemSol-ving_t	0.00	0.07	0.00	-0.07	0.00	1.00	0.00	-0.13	0.00	0.05	0.00	0.05	0.00	0.08	0.00
BP_Cogn-BehProb-lemSol-ving_t	0.15	0.00	-0.03	0.00	0.47	0.00	1.00	0.00	-0.01	0.00	0.27	0.00	0.19	0.00	0.19
WP_Supp-ression-Distrac-tion_t	0.01	0.27	0.00	-0.10	0.00	-0.13	0.00	1.00	0.00	-0.06	0.00	-0.06	0.00	-0.25	0.00
BP_Supp-ression-Distrac-tion_t	0.20	0.00	0.39	0.00	0.05	0.00	-0.01	0.00	1.00	0.00	0.18	0.00	0.01	0.00	-0.19
WP_Bo-dySocial_t	-0.04	-0.04	0.00	0.09	0.00	0.05	0.00	-0.06	0.00	1.00	0.00	0.07	0.00	0.15	0.00

Predictor	Stress_t-1	WP_StateRum_t	BP_StateRum_t	WP_Perspective Change_t	BP_Perspective Change_t	WP_CognBehProblemSolving_t	BP_CognBehProblemSolving_t	WP_Suppression-Distraction_t	BP_Suppression-Distraction_t	WP_BodySocial_t	BP_BodySocial_t	WP Self-efficacy_t	BP Self-efficacy_t	WP_SuccessEmotionRegulation _t	BP_SuccessEmotionRegulation _t
BP_BodySocial_t	0.10	0.00	0.12	0.00	0.35	0.00	0.27	0.00	0.18	0.00	1.00	0.00	0.14	0.00	0.22
WP Self-efficacy_t	-0.04	-0.19	0.00	0.17	0.00	0.05	0.00	-0.06	0.00	0.07	0.00	1.00	0.00	0.23	0.00
BP Self-efficacy_t	0.00	0.00	-0.06	0.00	0.26	0.00	0.19	0.00	0.01	0.00	0.14	0.00	1.00	0.00	0.32
WP_SuccessEmotionRegulation _t	-0.09	-0.38	0.00	0.24	0.00	0.08	0.00	-0.25	0.00	0.15	0.00	0.23	0.00	1.00	0.00
BP_SuccessEmotionRegulation _t	-0.09	0.00	-0.37	0.00	0.33	0.00	0.19	0.00	-0.19	0.00	0.22	0.00	0.32	0.00	1.00

Correlation Matrix of Predictors Model 4: state-rumination_t

Predictor	StateRum_ t-1	WP_Stress _t	BP_Stress _t	WP_Per-spec-tiveChange _t	BP_Per-spec-tiveChange _t	WP_Cogn-BehProb-lemSol-ving_t	BP_Cogn-BehProb-lemSol-ving_t	WP_Supp-ression-Distrac-tion_t	BP_Supp-ression-Distrac-tion_t	WP_Bo-dySocial_t	BP_Bo-dySocial_t	WP Self-efficacy_t	BP Self-efficacy_t	WP_Suc-cessEmoti-onRegula-tion _t	BP_Suc-cessEmoti-onRegula-tion _t
StateRum_ t-1	1.00	0.08	0.26	-0.01	0.00	-0.02	0.02	0.07	0.22	0.00	0.08	-0.05	-0.02	-0.07	-0.17
WP_Stress _t	0.08	1.00	0.00	-0.10	0.00	0.17	0.00	0.17	0.00	-0.06	0.00	-0.06	0.00	-0.39	0.00
BP_Stress _t	0.26	0.00	1.00	0.00	-0.04	0.00	0.28	0.00	0.31	0.00	0.12	0.00	0.07	0.00	-0.27
WP_Per-spec-tiveChange _t	-0.01	-0.10	0.00	1.00	0.00	-0.07	0.00	-0.10	0.00	0.09	0.00	0.17	0.00	0.24	0.00
BP_Per-spec-tiveChange _t	0.00	0.00	-0.04	0.00	1.00	0.00	0.47	0.00	0.05	0.00	0.35	0.00	0.26	0.00	0.33
WP_Cogn-BehProb-lemSol-ving_t	-0.02	0.17	0.00	-0.07	0.00	1.00	0.00	-0.13	0.00	0.05	0.00	0.05	0.00	0.08	0.00
BP_Cogn-BehProb-lemSol-ving_t	0.02	0.00	0.28	0.00	0.47	0.00	1.00	0.00	-0.01	0.00	0.27	0.00	0.19	0.00	0.19
WP_Supp-ression-Distrac-tion_t	0.07	0.17	0.00	-0.10	0.00	-0.13	0.00	1.00	0.00	-0.06	0.00	-0.06	0.00	-0.25	0.00
BP_Supp-ression-Distrac-tion_t	0.22	0.00	0.31	0.00	0.05	0.00	-0.01	0.00	1.00	0.00	0.18	0.00	0.01	0.00	-0.19
WP_Bo-dySocial_t	0.00	-0.06	0.00	0.09	0.00	0.05	0.00	-0.06	0.00	1.00	0.00	0.07	0.00	0.15	0.00

Predictor	StateRum_ t-1	WP_Stress _t	BP_Stress _t	WP_Per-spec-tiveChange _t	BP_Per-spec-tiveChange _t	WP_Cogn-BehProb-lemSol-ving_t	BP_Cogn-BehProb-lemSol-ving_t	WP_Supp-ression-Distrac-tion_t	BP_Supp-ression-Distrac-tion_t	WP_Bo-dySocial_t	BP_Bo-dySocial_t	WP Self-efficacy_t	BP Self-efficacy_t	WP_Suc-cessEmoti-onRegula-tion _t	BP_Suc-cessEmoti-onRegula-tion _t
BP_Bo-dySocial_t	0.08	0.00	0.12	0.00	0.35	0.00	0.27	0.00	0.18	0.00	1.00	0.00	0.14	0.00	0.22
WP Self-efficacy_t	-0.05	-0.06	0.00	0.17	0.00	0.05	0.00	-0.06	0.00	0.07	0.00	1.00	0.00	0.23	0.00
BP Self-efficacy_t	-0.02	0.00	0.07	0.00	0.26	0.00	0.19	0.00	0.01	0.00	0.14	0.00	1.00	0.00	0.32
WP_Suc-cessEmoti-onRegula-tion _t	-0.07	-0.39	0.00	0.24	0.00	0.08	0.00	-0.25	0.00	0.15	0.00	0.23	0.00	1.00	0.00
BP_Suc-cessEmoti-onRegula-tion _t	-0.17	0.00	-0.27	0.00	0.33	0.00	0.19	0.00	-0.19	0.00	0.22	0.00	0.32	0.00	1.00

Supplementary material S6: Prevalence of psychiatric disorders in the sample

Absolute and relative frequencies of self-reported primary ICD-10 diagnoses

ICD-10 diagnosis	F21	F32	F33	F40	F43.1	F43.2	F50	F60
n	1	13	1	6	2	1	7	2
%	3.03 %	39.39 %	3.03 %	18.18 %	6.06 %	3.03 %	21.21 %	6.06 %

Absolute and relative frequencies of self-reported secondary ICD-10 diagnoses

ICD-10 diagnosis	F20	F32	F40	F42	F43.1	F60
n	1	9	3	1	1	1
%	6.25 %	56.25 %	18.75 %	6.25 %	6.25 %	6.25 %

Supplementary material S7: Oblimin rotated factor loadings of the exploratory multilevel factor analysis (Between Level Results)

	1	2	3	4
cognitive problem-solving	1.375			
behavioral problem-solving	0.508	-0.514		-0.494
reappraisal		0.875		
selfcompassion		1.145		
acceptance		0.904		
mindfulness		0.536		0.463
distraction			1.175	
suppression			0.487	
social support				0.665
body-based				0.898

Note. Factor loadings < .4 are not displayed in terms of clearer visualization.

Supplementary material S8: MPlus output

Mplus VERSION 8.9
MUTHEN & MUTHEN
07/04/2023 8:38 AM

OUTPUT SECTIONS

```
Input Instructions
Input Warnings And Errors
Summary Of Analysis
Summary Of Data
Univariate Proportions And Counts For Categorical Variables
Sample Statistics
Results For Exploratory Factor Analysis
- for 2 within factor(s) and 4 between factor(s)
- for 2 within factor(s) and 4 between factor(s)
Plot Information
```

INPUT INSTRUCTIONS

```
  TITLE:  EFA

  DATA:
    FILE IS "C:\Users\Psychophysiologie\Desktop\EMA_data2.csv";

  VARIABLE:
    NAMES ARE ID KognitivPL BehavioralPL Umbewerten
    Selfcompassion Akzeptanz Mindfulness Distraction Suppression Social
    Body;
    USEVARIABLES ARE KognitivPL BehavioralPL Umbewerten Selfcompassion
    Akzeptanz Mindfulness Distraction Suppression Social Body;
    CATEGORICAL ARE KognitivPL-Body;
    CLUSTER = ID;


  Analysis:
      Type = twolevel efa 2 2 4 4;

      estimator=wlsmv;

      ROTATION = oblimin;



    PLOT:

      Type = plot2;



    OUTPUT:

      sampstat;




*** WARNING in VARIABLE command
  Note that only the first 8 characters of variable names are used in the output.
  Shorten variable names to avoid any confusion.
*** WARNING in PLOT command
  Note that only the first 8 characters of variable names are used in plots.
  If variable names are not unique within the first 8 characters, problems
  may occur.
   2 WARNING(S) FOUND IN THE INPUT INSTRUCTIONS



EFA
```

SUMMARY OF ANALYSIS

```
Number of groups                                                 1
Number of observations                                         356

Number of dependent variables                                   10
```

```
Number of independent variables                                  0
Number of continuous latent variables                            0

Observed dependent variables

  Binary and ordered categorical (ordinal)
   KOGNITIV    BEHAVIOR    UMBEWERT    SELFCOMP    AKZEPTAN    MINDFULN
   DISTRACT    SUPPRESS    SOCIAL      BODY

Variables with special functions

  Cluster variable      ID

Estimator                                                    WLSMV
Rotation                                                  OBLIMIN
Row standardization                                   CORRELATION
Type of rotation                                          OBLIQUE
Gamma value                                             0.000D+00
Optimization Specifications for the Quasi-Newton Algorithm for
Continuous Outcomes
  Maximum number of iterations                                1000
  Convergence criterion                                  0.100D-05
Optimization Specifications for the EM Algorithm
  Maximum number of iterations                                 500
  Convergence criteria
    Loglikelihood change                                 0.100D-02
    Relative loglikelihood change                        0.100D-05
    Derivative                                           0.100D-02
Optimization Specifications for the M step of the EM Algorithm for
Categorical Latent variables
  Number of M step iterations                                    1
  M step convergence criterion                           0.100D-02
  Basis for M step termination                           ITERATION
Optimization Specifications for the M step of the EM Algorithm for
Censored, Binary or Ordered Categorical (Ordinal), Unordered
Categorical (Nominal) and Count Outcomes
  Number of M step iterations                                    1
  M step convergence criterion                           0.100D-02
  Basis for M step termination                           ITERATION
  Maximum value for logit thresholds                            10
  Minimum value for logit thresholds                           -10
  Minimum expected cell size for chi-square              0.100D-01
Optimization Specifications for the Exploratory Factor Analysis
Rotation Algorithm
  Number of random starts                                       30
  Maximum number of iterations                               10000
  Derivative convergence criterion                       0.100D-04
Optimization algorithm                                          FS
Integration Specifications
  Type                                                    STANDARD
  Number of integration points                                   7
  Dimensions of numerical integration                            2
  Adaptive quadrature                                           ON
Link                                                        PROBIT
Cholesky                                                        ON

Input data file(s)
  C:\Users\Psychophysiologie\Desktop\EMA_data2.csv
Input data format  FREE
```

SUMMARY OF DATA

```
     Number of clusters                        38

     Average cluster size        9.368

     Estimated Intraclass Correlations for the Y Variables

               Intraclass              Intraclass              Intraclass
     Variable  Correlation   Variable  Correlation   Variable  Correlation

     KOGNITIV     0.166      BEHAVIOR     0.160      UMBEWERT     0.118
     SELFCOMP     0.018      AKZEPTAN     0.069      MINDFULN     0.157
     DISTRACT     0.214      SUPPRESS     0.198      SOCIAL       0.106
     BODY         0.100
```

UNIVARIATE PROPORTIONS AND COUNTS FOR CATEGORICAL VARIABLES

```
    KOGNITIV
      Category 1    0.489         174.000
      Category 2    0.511         182.000
    BEHAVIOR
      Category 1    0.551         196.000
      Category 2    0.449         160.000
    UMBEWERT
      Category 1    0.629         224.000
      Category 2    0.371         132.000
    SELFCOMP
      Category 1    0.494         176.000
      Category 2    0.506         180.000
    AKZEPTAN
      Category 1    0.492         175.000
      Category 2    0.508         181.000
    MINDFULN
      Category 1    0.579         206.000
      Category 2    0.421         150.000
    DISTRACT
      Category 1    0.610         217.000
      Category 2    0.390         139.000
    SUPPRESS
      Category 1    0.646         230.000
      Category 2    0.354         126.000
    SOCIAL
      Category 1    0.654         233.000
      Category 2    0.346         123.000
    BODY
      Category 1    0.702         250.000
      Category 2    0.298         106.000
```

SAMPLE STATISTICS

ESTIMATED SAMPLE STATISTICS

MEANS/INTERCEPTS/THRESHOLDS

KOGNITIV	BEHAVIOR	UMBEWERT	SELFCOMP	AKZEPTAN
-0.015	0.152	0.342	-0.016	-0.028

MEANS/INTERCEPTS/THRESHOLDS

MINDFULN	DISTRACT	SUPPRESS	SOCIAL$1	BODY$1
0.204	0.324	0.401	0.425	0.551

WITHIN LEVEL VARIANCE/COVARIANCE

	KOGNITIV	BEHAVIOR	UMBEWERT	SELFCOMP	AKZEPTAN
KOGNITIV	1.000				
BEHAVIOR	0.794	1.000			
UMBEWERT	0.573	0.571	1.000		
SELFCOMP	0.524	0.533	0.634	1.000	
AKZEPTAN	0.592	0.486	0.643	0.714	1.000
MINDFULN	0.602	0.374	0.634	0.650	0.693
DISTRACT	0.407	0.085	0.297	0.321	0.522
SUPPRESS	0.455	0.355	0.219	0.337	0.368
SOCIAL	0.486	0.373	0.468	0.463	0.368
BODY	0.393	0.408	0.378	0.404	0.486

WITHIN LEVEL VARIANCE/COVARIANCE

	MINDFULN	DISTRACT	SUPPRESS	SOCIAL	BODY
MINDFULN	1.000				
DISTRACT	0.426	1.000			
SUPPRESS	0.259	0.491	1.000		
SOCIAL	0.561	0.283	0.143	1.000	

BODY	0.516	0.551	0.229	0.258	1.000

WITHIN LEVEL CORRELATION

	KOGNITIV	BEHAVIOR	UMBEWERT	SELFCOMP	AKZEPTAN
KOGNITIV	1.000				
BEHAVIOR	0.794	1.000			
UMBEWERT	0.573	0.571	1.000		
SELFCOMP	0.524	0.533	0.634	1.000	
AKZEPTAN	0.592	0.486	0.643	0.714	1.000
MINDFULN	0.602	0.374	0.634	0.650	0.693
DISTRACT	0.407	0.085	0.297	0.321	0.522
SUPPRESS	0.455	0.355	0.219	0.337	0.368
SOCIAL	0.486	0.373	0.468	0.463	0.368
BODY	0.393	0.408	0.378	0.404	0.486

WITHIN LEVEL CORRELATION

	MINDFULN	DISTRACT	SUPPRESS	SOCIAL	BODY
MINDFULN	1.000				
DISTRACT	0.426	1.000			
SUPPRESS	0.259	0.491	1.000		
SOCIAL	0.561	0.283	0.143	1.000	
BODY	0.516	0.551	0.229	0.258	1.000

BETWEEN LEVEL VARIANCE/COVARIANCE

	KOGNITIV	BEHAVIOR	UMBEWERT	SELFCOMP	AKZEPTAN
KOGNITIV	0.199				
BEHAVIOR	0.167	0.191			
UMBEWERT	-0.089	-0.159	0.133		
SELFCOMP	-0.003	-0.026	0.042	0.019	
AKZEPTAN	-0.010	-0.080	0.078	0.037	0.075
MINDFULN	-0.062	-0.111	0.112	0.042	0.065
DISTRACT	-0.079	-0.090	0.130	0.023	-0.003
SUPPRESS	-0.001	-0.018	0.041	0.025	0.002
SOCIAL	0.006	-0.029	-0.023	-0.027	-0.005
BODY	0.012	-0.056	0.026	-0.025	0.012

BETWEEN LEVEL VARIANCE/COVARIANCE

	MINDFULN	DISTRACT	SUPPRESS	SOCIAL	BODY
MINDFULN	0.186				
DISTRACT	0.075	0.273			
SUPPRESS	-0.021	0.133	0.247		
SOCIAL	0.005	0.044	-0.031	0.119	
BODY	0.069	0.051	-0.015	0.072	0.111

BETWEEN LEVEL CORRELATION

	KOGNITIV	BEHAVIOR	UMBEWERT	SELFCOMP	AKZEPTAN
KOGNITIV	1.000				
BEHAVIOR	0.858	1.000			
UMBEWERT	-0.549	-1.000	1.000		
SELFCOMP	-0.043	-0.434	0.846	1.000	
AKZEPTAN	-0.084	-0.670	0.787	0.997	1.000
MINDFULN	-0.322	-0.588	0.714	0.724	0.550
DISTRACT	-0.340	-0.394	0.679	0.320	-0.023
SUPPRESS	-0.003	-0.083	0.228	0.374	0.011
SOCIAL	0.037	-0.194	-0.182	-0.576	-0.054
BODY	0.081	-0.388	0.216	-0.560	0.137

BETWEEN LEVEL CORRELATION

	MINDFULN	DISTRACT	SUPPRESS	SOCIAL	BODY
MINDFULN	1.000				
DISTRACT	0.334	1.000			
SUPPRESS	-0.097	0.512	1.000		
SOCIAL	0.034	0.245	-0.180	1.000	
BODY	0.483	0.294	-0.091	0.628	1.000

RESULTS FOR EXPLORATORY FACTOR ANALYSIS

EIGENVALUES FOR WITHIN LEVEL SAMPLE CORRELATION MATRIX

1	2	3	4	5
5.172	1.194	0.972	0.708	0.650

EIGENVALUES FOR WITHIN LEVEL SAMPLE CORRELATION MATRIX

6	7	8	9	10
0.399	0.344	0.307	0.199	0.055

EIGENVALUES FOR BETWEEN LEVEL SAMPLE CORRELATION MATRIX

1	2	3	4	5
4.464	2.342	1.485	1.213	0.595

EIGENVALUES FOR BETWEEN LEVEL SAMPLE CORRELATION MATRIX

6	7	8	9	10
0.424	0.242	-0.091	-0.210	-0.464

EXPLORATORY FACTOR ANALYSIS WITH 2 WITHIN FACTOR(S) AND 4 BETWEEN FACTOR(S):

MODEL FIT INFORMATION

```
Number of Free Parameters                       73

Chi-Square Test of Model Fit

          Value                             79.198*
          Degrees of Freedom                    37
          P-Value                           0.0001

*   The chi-square value for MLM, MLMV, MLR, ULSMV, WLSM and WLSMV cannot be used
    for chi-square difference testing in the regular way.  MLM, MLR and WLSM
    chi-square difference testing is described on the Mplus website.  MLMV, WLSMV,
    and ULSMV difference testing is done using the DIFFTEST option.

RMSEA (Root Mean Square Error Of Approximation)

          Estimate                           0.057
          90 Percent C.I.                    0.039  0.074
          Probability RMSEA <= .05           0.248

CFI/TLI

          CFI                                0.967
          TLI                                0.919

Chi-Square Test of Model Fit for the Baseline Model

          Value                           1356.250
          Degrees of Freedom                    90
          P-Value                           0.0000

SRMR (Standardized Root Mean Square Residual)

          Value for Within                   0.075
          Value for Between                  0.074


MINIMUM ROTATION FUNCTION VALUE       0.07617
```

WITHIN LEVEL RESULTS

```
           OBLIMIN ROTATED LOADINGS (* significant at 5% level)
                  1             2
              ________      ________
 KOGNITIV       0.582*        0.391*
 BEHAVIOR       1.001*       -0.003
 UMBEWERT       0.277*        0.578*
 SELFCOMP       0.195*        0.663*
 AKZEPTAN       0.070         0.816*
 MINDFULN       0.007         0.826*
 DISTRACT      -0.282*        0.767*
 SUPPRESS       0.124         0.376*
 SOCIAL         0.171         0.444*
 BODY          -0.017         0.615*


           OBLIMIN FACTOR CORRELATIONS (* significant at 5% level)
                  1             2
              ________      ________
      1         1.000
      2         0.510*        1.000


           ESTIMATED RESIDUAL VARIANCES
              KOGNITIV      BEHAVIOR      UMBEWERT      SELFCOMP      AKZEPTAN
              ________      ________      ________      ________      ________
                0.276         0.000         0.425         0.390         0.272


           ESTIMATED RESIDUAL VARIANCES
              MINDFULN      DISTRACT      SUPPRESS      SOCIAL        BODY
              ________      ________      ________      ________      ________
                0.311         0.553         0.795         0.697         0.631


           S.E. OBLIMIN ROTATED LOADINGS
                  1             2
              ________      ________
 KOGNITIV       0.124         0.112
 BEHAVIOR       0.080         0.031
 UMBEWERT       0.081         0.067
 SELFCOMP       0.083         0.059
 AKZEPTAN       0.075         0.060
 MINDFULN       0.068         0.052
 DISTRACT       0.046         0.069
 SUPPRESS       0.083         0.077
 SOCIAL         0.107         0.092
 BODY           0.092         0.073


           S.E. OBLIMIN FACTOR CORRELATIONS
                  1             2
              ________      ________
      1         0.000
      2         0.064         0.000


           S.E. ESTIMATED RESIDUAL VARIANCES
              KOGNITIV      BEHAVIOR      UMBEWERT      SELFCOMP      AKZEPTAN
              ________      ________      ________      ________      ________
                0.079         0.182         0.046         0.051         0.059


           S.E. ESTIMATED RESIDUAL VARIANCES
              MINDFULN      DISTRACT      SUPPRESS      SOCIAL        BODY
              ________      ________      ________      ________      ________
                0.050         0.082         0.052         0.074         0.052


           Est./S.E. OBLIMIN ROTATED LOADINGS
                  1             2
              ________      ________
 KOGNITIV       4.680         3.494
 BEHAVIOR      12.540        -0.089
 UMBEWERT       3.437         8.575
 SELFCOMP       2.350        11.196
 AKZEPTAN       0.927        13.635
```

MINDFULN	0.104	15.765
DISTRACT	-6.074	11.037
SUPPRESS	1.489	4.858
SOCIAL	1.600	4.846
BODY	-0.183	8.462

Est./S.E. OBLIMIN FACTOR CORRELATIONS

	1	2
1	0.000	
2	8.009	0.000

Est./S.E. ESTIMATED RESIDUAL VARIANCES

KOGNITIV	BEHAVIOR	UMBEWERT	SELFCOMP	AKZEPTAN
3.495	0.001	9.154	7.714	4.641

Est./S.E. ESTIMATED RESIDUAL VARIANCES

MINDFULN	DISTRACT	SUPPRESS	SOCIAL	BODY
6.254	6.764	15.331	9.444	12.166

FACTOR STRUCTURE

	1	2
KOGNITIV	0.781	0.688
BEHAVIOR	1.000	0.507
UMBEWERT	0.572	0.720
SELFCOMP	0.533	0.763
AKZEPTAN	0.485	0.851
MINDFULN	0.428	0.830
DISTRACT	0.109	0.623
SUPPRESS	0.316	0.439
SOCIAL	0.397	0.531
BODY	0.297	0.607

ESTIMATED MODEL AND RESIDUALS (OBSERVED - ESTIMATED)

Model Estimated Correlations

	KOGNITIV	BEHAVIOR	UMBEWERT	SELFCOMP	AKZEPTAN
KOGNITIV	1.000				
BEHAVIOR	0.781	1.000			
UMBEWERT	0.614	0.571	1.000		
SELFCOMP	0.609	0.532	0.589	1.000	
AKZEPTAN	0.615	0.484	0.627	0.659	1.000
MINDFULN	0.574	0.426	0.599	0.634	0.707
DISTRACT	0.307	0.107	0.391	0.434	0.516
SUPPRESS	0.356	0.315	0.342	0.353	0.381
SOCIAL	0.439	0.396	0.417	0.429	0.461
BODY	0.410	0.296	0.433	0.460	0.516

Model Estimated Correlations

	MINDFULN	DISTRACT	SUPPRESS	SOCIAL	BODY
MINDFULN	1.000				
DISTRACT	0.516	1.000			
SUPPRESS	0.365	0.248	1.000		
SOCIAL	0.441	0.295	0.249	1.000	
BODY	0.504	0.382	0.265	0.320	1.000

Residuals for Correlations

	KOGNITIV	BEHAVIOR	UMBEWERT	SELFCOMP	AKZEPTAN
KOGNITIV	0.000				
BEHAVIOR	0.013	0.000			
UMBEWERT	-0.042	0.000	0.000		
SELFCOMP	-0.085	0.002	0.045	0.000	
AKZEPTAN	-0.023	0.003	0.016	0.055	0.000

MINDFULN	0.028	-0.052	0.035	0.016	-0.014
DISTRACT	0.100	-0.022	-0.093	-0.113	0.006
SUPPRESS	0.100	0.040	-0.123	-0.016	-0.012
SOCIAL	0.047	-0.023	0.051	0.034	-0.092
BODY	-0.017	0.112	-0.055	-0.056	-0.029

Residuals for Correlations

	MINDFULN	DISTRACT	SUPPRESS	SOCIAL	BODY
MINDFULN	0.000				
DISTRACT	-0.090	0.000			
SUPPRESS	-0.106	0.243	0.000		
SOCIAL	0.120	-0.012	-0.106	0.000	
BODY	0.013	0.169	-0.036	-0.062	0.000

EXPLORATORY FACTOR ANALYSIS WITH 2 WITHIN FACTOR(S) AND 4 BETWEEN FACTOR(S):

MINIMUM ROTATION FUNCTION VALUE 0.47225

BETWEEN LEVEL RESULTS

OBLIMIN ROTATED LOADINGS (* significant at 5% level)

	1	2	3	4
KOGNITIV	1.375	0.050	-0.004	0.072
BEHAVIOR	0.508	-0.514	-0.056	-0.494
UMBEWERT	-0.225	0.875	0.280	0.223
SELFCOMP	0.113	1.145	0.104	-0.377
AKZEPTAN	0.043	0.904	-0.251	0.314
MINDFULN	-0.105	0.536	-0.019	0.463
DISTRACT	-0.010	0.016	1.175*	0.059
SUPPRESS	0.067	0.088	0.487	-0.275
SOCIAL	0.058	-0.386	0.136	0.665
BODY	0.169	-0.086	0.158	0.898

OBLIMIN FACTOR CORRELATIONS (* significant at 5% level)

	1	2	3	4
1	1.000			
2	-0.173	1.000		
3	-0.202	0.203	1.000	
4	-0.151	0.013	0.151	1.000

ESTIMATED RESIDUAL VARIANCES

KOGNITIV	BEHAVIOR	UMBEWERT	SELFCOMP	AKZEPTAN
-0.846	0.026	-0.176	-0.466	0.141

ESTIMATED RESIDUAL VARIANCES

MINDFULN	DISTRACT	SUPPRESS	SOCIAL	BODY
0.454	-0.418	0.708	0.394	0.149

S.E. OBLIMIN ROTATED LOADINGS

	1	2	3	4
KOGNITIV	1.647	0.393	0.105	0.446
BEHAVIOR	1.168	1.729	0.276	1.461
UMBEWERT	0.660	0.739	0.264	2.113
SELFCOMP	0.479	1.979	0.401	2.079
AKZEPTAN	0.333	1.222	0.257	2.138
MINDFULN	0.501	1.552	0.228	1.308
DISTRACT	0.105	0.344	0.471	0.398
SUPPRESS	0.380	1.172	0.298	0.314
SOCIAL	0.186	2.129	0.508	0.985

BODY	0.164	2.640	0.367	0.663

S.E. OBLIMIN FACTOR CORRELATIONS

	1	2	3	4
1	0.000			
2	0.292	0.000		
3	0.313	0.650	0.000	
4	0.364	0.775	0.688	0.000

S.E. ESTIMATED RESIDUAL VARIANCES

KOGNITIV	BEHAVIOR	UMBEWERT	SELFCOMP	AKZEPTAN
4.646	0.553	0.378	2.238	0.585

S.E. ESTIMATED RESIDUAL VARIANCES

MINDFULN	DISTRACT	SUPPRESS	SOCIAL	BODY
0.255	1.001	0.245	0.584	0.827

Est./S.E. OBLIMIN ROTATED LOADINGS

	1	2	3	4
KOGNITIV	0.835	0.127	-0.043	0.162
BEHAVIOR	0.434	-0.297	-0.205	-0.338
UMBEWERT	-0.340	1.185	1.058	0.105
SELFCOMP	0.237	0.579	0.260	-0.181
AKZEPTAN	0.128	0.740	-0.979	0.147
MINDFULN	-0.209	0.346	-0.083	0.354
DISTRACT	-0.091	0.045	2.496	0.147
SUPPRESS	0.177	0.075	1.636	-0.876
SOCIAL	0.314	-0.181	0.267	0.675
BODY	1.032	-0.033	0.432	1.355

Est./S.E. OBLIMIN FACTOR CORRELATIONS

	1	2	3	4
1	0.000			
2	-0.594	0.000		
3	-0.645	0.313	0.000	
4	-0.415	0.016	0.220	0.000

Est./S.E. ESTIMATED RESIDUAL VARIANCES

KOGNITIV	BEHAVIOR	UMBEWERT	SELFCOMP	AKZEPTAN
-0.182	0.048	-0.466	-0.208	0.241

Est./S.E. ESTIMATED RESIDUAL VARIANCES

MINDFULN	DISTRACT	SUPPRESS	SOCIAL	BODY
1.779	-0.418	2.886	0.676	0.181

FACTOR STRUCTURE

	1	2	3	4
KOGNITIV	1.356	-0.188	-0.261	-0.136
BEHAVIOR	0.683	-0.620	-0.338	-0.586
UMBEWERT	-0.467	0.974	0.537	0.310
SELFCOMP	-0.049	1.142	0.257	-0.364
AKZEPTAN	-0.111	0.850	-0.028	0.281
MINDFULN	-0.264	0.556	0.181	0.482
DISTRACT	-0.258	0.257	1.189	0.238
SUPPRESS	-0.004	0.172	0.450	-0.210
SOCIAL	-0.003	-0.360	0.146	0.672
BODY	0.017	-0.072	0.242	0.895

ESTIMATED MODEL AND RESIDUALS (OBSERVED - ESTIMATED)

Model Estimated Correlations

	KOGNITIV	BEHAVIOR	UMBEWERT	SELFCOMP	AKZEPTAN
KOGNITIV	1.000				
BEHAVIOR	0.867	1.000			
UMBEWERT	-0.573	-0.921	1.000		
SELFCOMP	-0.038	-0.447	1.001	1.000	
AKZEPTAN	-0.090	-0.630	0.823	0.852	1.000
MINDFULN	-0.301	-0.669	0.705	0.444	0.598
DISTRACT	-0.330	-0.448	0.669	0.299	-0.003
SUPPRESS	-0.015	-0.012	0.230	0.323	-0.024
SOCIAL	0.027	-0.156	-0.124	-0.651	-0.152
BODY	0.083	-0.410	0.200	-0.393	0.156

Model Estimated Correlations

	MINDFULN	DISTRACT	SUPPRESS	SOCIAL	BODY
MINDFULN	1.000				
DISTRACT	0.252	1.000			
SUPPRESS	-0.013	0.519	1.000		
SOCIAL	0.115	0.205	-0.145	1.000	
BODY	0.369	0.336	-0.133	0.657	1.000

Residuals for Correlations

	KOGNITIV	BEHAVIOR	UMBEWERT	SELFCOMP	AKZEPTAN
KOGNITIV	0.000				
BEHAVIOR	-0.009	0.000			
UMBEWERT	0.023	-0.079	0.000		
SELFCOMP	-0.005	0.013	-0.155	0.000	
AKZEPTAN	0.006	-0.040	-0.037	0.146	0.000
MINDFULN	-0.021	0.081	0.009	0.280	-0.048
DISTRACT	-0.010	0.053	0.011	0.021	-0.020
SUPPRESS	0.011	-0.071	-0.003	0.051	0.035
SOCIAL	0.010	-0.038	-0.058	0.075	0.098
BODY	-0.002	0.022	0.015	-0.167	-0.018

Residuals for Correlations

	MINDFULN	DISTRACT	SUPPRESS	SOCIAL	BODY
MINDFULN	0.000				
DISTRACT	0.082	0.000			
SUPPRESS	-0.084	-0.007	0.000		
SOCIAL	-0.082	0.040	-0.034	0.000	
BODY	0.114	-0.042	0.042	-0.029	0.000

PLOT INFORMATION

The following plots are available:

  Eigenvalues for exploratory factor analysis
  Sample proportions

DIAGRAM INFORMATION

  Mplus diagrams are currently not available for multilevel analysis.
  No diagram output was produced.

```
    Beginning Time:  08:38:29
       Ending Time:  08:38:35
      Elapsed Time:  00:00:06
```

MUTHEN & MUTHEN
3463 Stoner Ave.
Los Angeles, CA 90066

Tel: (310) 391-9971
Fax: (310) 391-8971
Web: www.StatModel.com

Support: Support@StatModel.com

Copyright (c) 1998-2023 Muthen & Muthen

Supplementary material S9: Analysis using PCA including all data entries

Performing a principal component analysis (PCA) using varimax-rotation with Kaiser-Normalization, four principal components were extracted according to the Scree-Plot and the Kaiser-Guttman-criterion. The rotation converged after 5 iterations. Clusters averaged the use of the following ERS: Cluster 1 consisted of self-compassion, acceptance, reframing and mindfulness, cluster 2 included cognitive and behavioral problem-solving, cluster 3 included suppression and distraction and cluster 4 included body-based and social regulation. We named the four clusters as follows: cognitive perspective change, cognitive-behavioral problem-solving, suppression-distraction, body-social. As the PCA analysis was performed on the basis of all data points (several per subjects), we replicated these findings in 5 randomly selected datasets only including one data point per subject.

Factor loadings of the Principal Component Analysis using all data entries

	PC1	PC2	PC3	PC4
self compassion	0.711			
acceptance	0.689			
reframing	0.665			
mindfulness	0.589			
cognitive problem-solving		0.797		
behavioral problem-solving		0.785		
suppression			0.810	
distraction			0.705	
body-based				0.786
social support				0.598

Note. Factor loadings smaller than +/-.3 are not displayed in terms of clearer visualization. PC = principal component.

Factor loadings of the Principal Component Analysis using 5 randomly chosen single data entries

data entry 1

	PC1	PC2	PC3	PC4
self compassion	.794			
acceptance	.725		.346	
reframing	.764			
mindfulness	.600			.529
cognitive problem-solving	.514	.664		
behavioral problem-solving	.469	.655		
suppression			.754	
distraction			.851	
body-based				.894
social support		.829		

data entry 2

	PC1	PC2	PC3	PC4
self compassion	.561	.547		
acceptance	.770			
reframing	.579	.390		
mindfulness	.841			
cognitive problem-solving		.850		
behavioral problem-solving		.820		
suppression			.820	
distraction			.801	.384
body-based				.816
social support	.383		.448	-.496

data entry 3

	PC1	PC2	PC3	PC4
self compassion	.756			
acceptance	.714	.359		
reframing	.606	.579		
mindfulness	.848			
cognitive problem-solving	.414	.711		
behavioral problem-solving		.889		
suppression			.810	
distraction	.380		.749	
body-based	.395		.323	
social support	.317			.713

data entry 4

	PC1	PC2	PC3	PC4
self compassion	.708	.388		
acceptance	.670	.375		
reframing	.693		.391	
mindfulness	.797			
cognitive problem-solving		.784		
behavioral problem-solving		.893		
suppression			.826	
distraction			.615	.637
body-based				.908
social support	.391	.410		

data entry 5

	PC1	PC2	PC3	PC4
self compassion	.768			
acceptance	.604	.353		
reframing	.592	.316	.433	
mindfulness	.774			
cognitive problem-solving		.870		
behavioral problem-solving		.857		
suppression			.859	
distraction			.698	.506
body-based				.866
social support	.431	.338		.445

Note. Factor loadings smaller than +/-.3 are not displayed in terms of clearer visualization. PC = principal component.

Supplementary material S10. Schematic illustration of the ERS summarized in scales. Please note, that "other" refers to the ERS entered via free-text which were not assigned to the four scales.

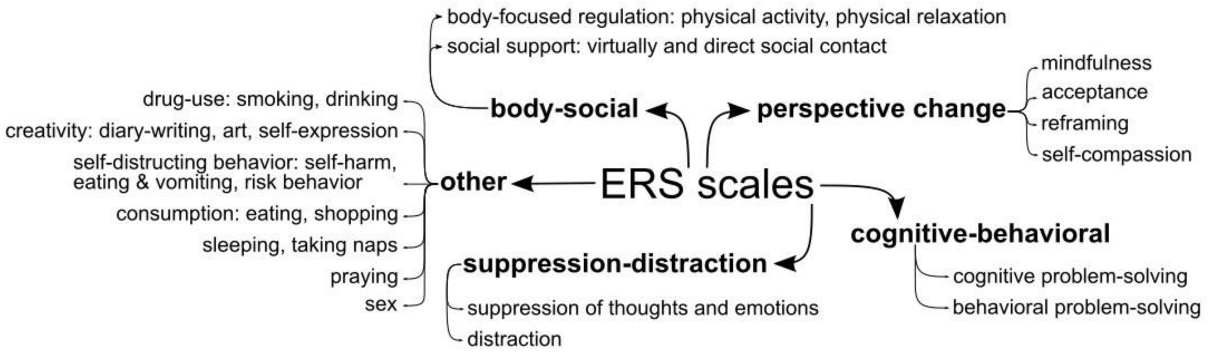

Supplementary material S11. Absolute frequencies of simultaneous ERS scale use and relative frequencies within ERS scale.

	used alone	used with one other scale	used with two other scales	used with three other scales	Σ
cognitive perspective change	20 (1.92 %)	217 (20.87 %)	433 (41.63 %)	370 (35.58 %)	1040 (100 %)
cognitive-behavioral problem-solving	16 (1.87 %)	121 (14.10 %)	351 (40.91 %)	370 (43.12 %)	858 (100 %)
suppression-distraction	28 (3.17 %)	165 (18.69 %)	320 (36.24 %)	370 (41.90 %)	883 (100 %)
body-social	11 (1.34 %)	93 (11.36 %)	345 (42.12 %)	370 (45.18 %)	819 (100 %)

Supplementary material S12. Absolute frequencies of ERS scale use.

		cognitive-behavioral problem-solving		suppression-distraction		body-social	
		no	yes	no	yes	no	yes
cognitive perspective change	no	78	108	42	144	71	115
	yes	290	750	301	739	336	704
cognitive-behavioral problem-solving	no			70	298	147	221
	yes			273	585	260	598
suppression-distraction	no					115	228
	yes					292	591

Supplementary material S13. Correlation matrix of between-subjects ERS scale use.

	cognitive-behavioral problem-solving	suppression-distraction	body-social
cognitive perspective change	r(142) = .444, p <.001	r(142) = .042, p = .620	r(142) = .313, p < .001
cognitive-behavioral problem-solving		r(142) = -.027, p = .746	r(142) = .246, p < .01
suppression-distraction			r(142) = .148, p = .076

Supplementary material S14. Correlation matrix of within-subjects ERS scale use.

	cognitive-behavioral problem-solving	suppression-distraction	body-social
cognitive perspective change	r(1081) = - .069, p < .05	r(1081) = - .098, p < .01	r(1081) = .086, p < .01
cognitive-behavioral problem-solving		r(1081) = .054, p = .076	r(1081) = - .128, p < .001
suppression-distraction			r(1081) = - .061, p < .05

References

Moher, D., Liberati, A., Tetzlaff, J., Altman, D.G., The PRISMA Group, 2009. Preferred Reporting Items for Systematic Reviews and Meta-Analyses: The PRISMA Statement. PLoS Med 6, e1000097. https://doi.org/10.1371/journal.pmed.1000097

Nolen-Hoeksema, S., 1991. Responses to depression and their effects on the duration of depressive episodes. Journal of Abnormal Psychology 100, 569–582. https://doi.org/10.1037/0021-843X.100.4.569

Szkodny, L.E., Newman, M.G., 2019. Delineating Characteristics of Maladaptive Repetitive Thought: Development and Preliminary Validation of the Perseverative Cognitions Questionnaire. Assessment 26, 1084–1104. https://doi.org/10.1177/1073191117698753
